# Supplementary material for: Cordycepin prevents radiation ulcer by inhibiting cell senescence via NRF2 and AMPK in rodents
Source: Nat Commun. 2019 Jun 10;10:2538. doi: 10.1038/s41467-019-10386-8 (PMC6557849; doi:10.1038/s41467-019-10386-8)
Supplement: Supplementary file 1 — Supplementary Information [file 41467_2019_10386_MOESM1_ESM.pdf]

Cordycepin prevents radiation ulcer by inhibiting cell senescence via NRF2 and AMPK in rodents

Wang *et al.*

Supplementary Figure 1.

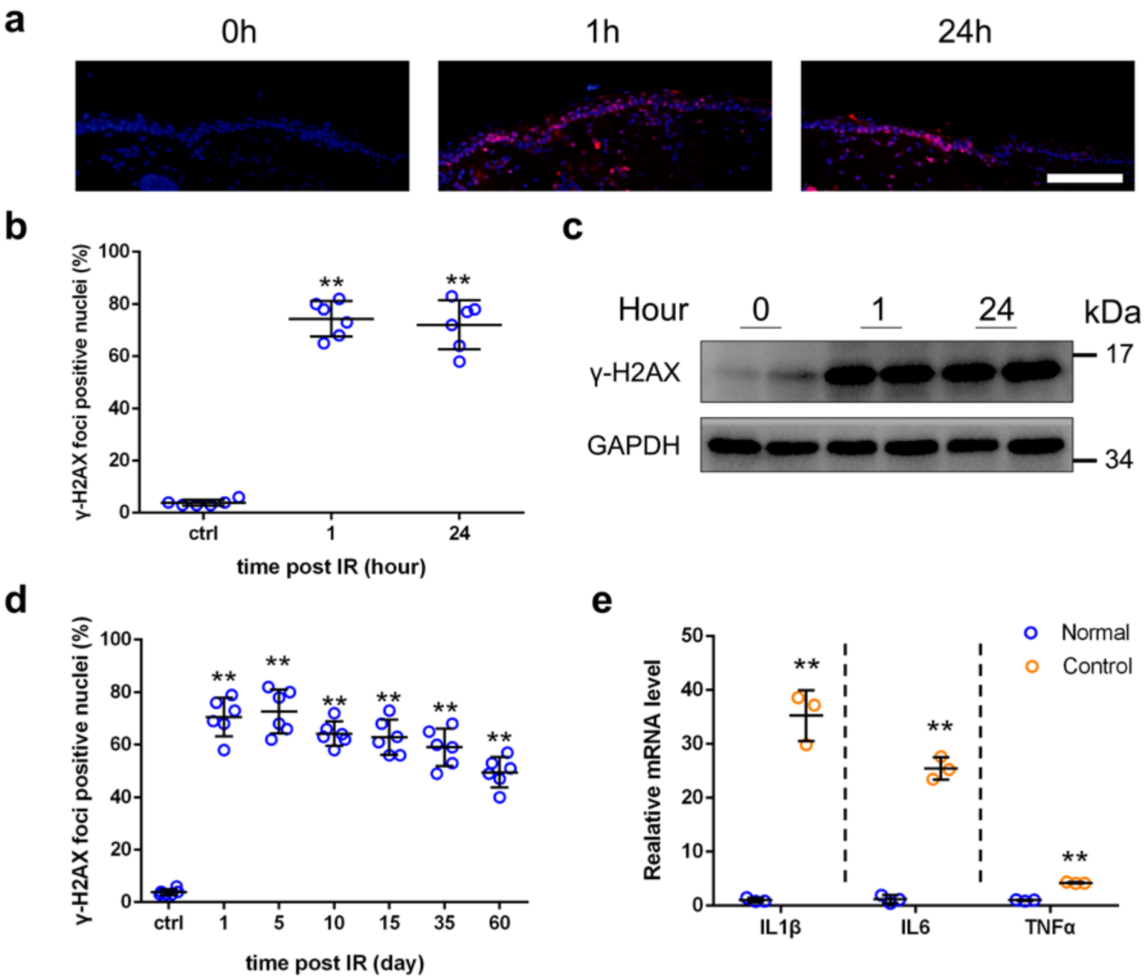

Supplementary Figure 1. γ-H2AX increased sharply within 1h after animals were irradiated. **(a)** Representative images of rat hind limb 0-24 hours post-radiation. **(b)** Quantification of γ-H2AX in rat skin tissues 0-24 hours post-radiation (n=6). **(c)** Western blot analysis of γ-H2AX expression in rat skin tissues 0-24 hours post-radiation. **(d)** Quantification of γ-H2AX in rat skin tissues 0-60 days post-radiation, the γ-H2AX positive nuclei was calculated using Image J software (imagej.nih.gov/ij/) (n=6). **(e)** Quantification of mRNA expression for IL1β, IL6 and TNFα in rat skin tissues 35 days post-radiation (n=3). Bars represent 50μm (a). Data in b, d and e represent the means ± S.D. (**P < 0.01; student's t-test)

Supplementary Figure 2.

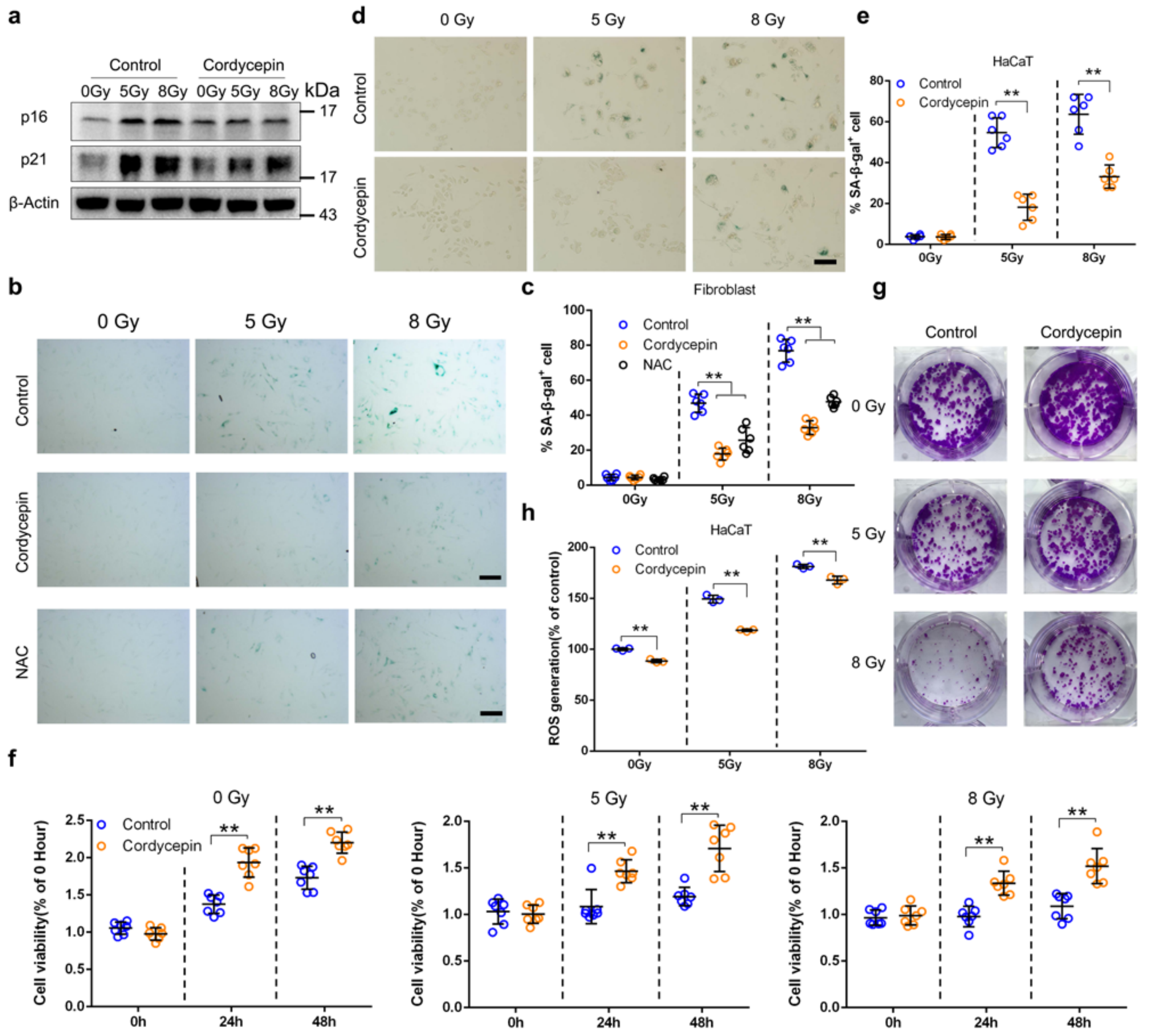

Supplementary Figure 2. Cordycepin decreases cell senescence and SASP in vitro. **(a)** Western blot analysis of p16 and p21 levels in irradiated control or cordycepin-treated HaCaT cells 7 days after radiation. **(b-c)** Staining and quantification for senescence-associated β-galactosidase (SA-β-gal) 7 days after radiation in fibroblasts pretreated with control, cordycepin or NAC (n=6). This test was repeated three times. Representative images were shown. **(d-e)** Staining and quantification for SA-β-gal 7 days after radiation in HaCaT cells pretreated or not (control) with cordycepin (n=6). This test was

repeated three times. Representative images were shown. **(f)** Representative proliferation of fibroblasts pretreated or not (control) with cordycepin following 0/5/8 Gy radiation (n=7). **(g)** Representative images of HaCaT cells colonies generated in survival assays following 0/5/8 Gy radiation. **(h)** Analysis of reactive oxygen species (ROS) levels by H2DCF-DA 24 hours after radiation in HaCaT cells pretreated or not (control) with cordycepin (n=3). Bars represent 250μm (b and d). Data in c, e, h and f represent the means ± S.D. (**P < 0.01; student's t-test)

Supplementary Figure 3.

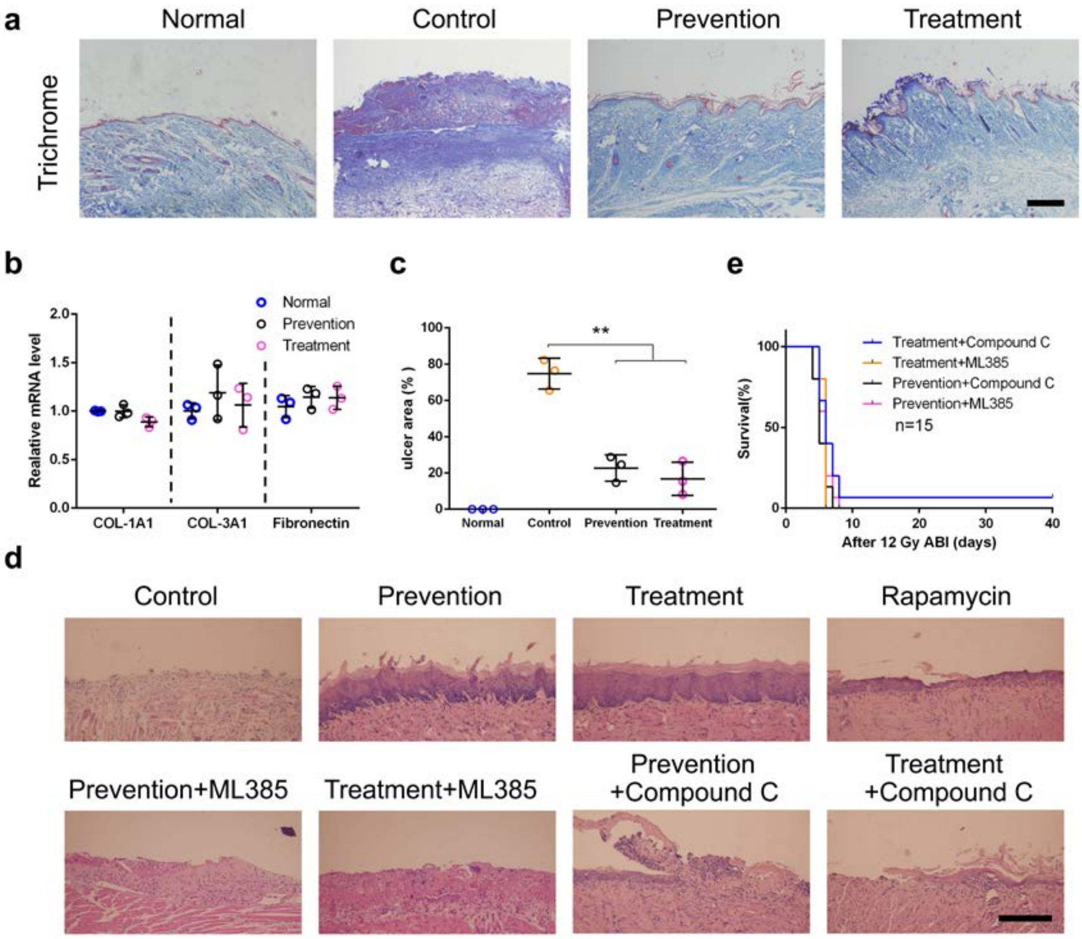

Supplementary Figure 3. Cordycepin prevents radiation ulcer. **(a)** Masson's Trichrome staining of the skins from normal rats and irradiated rats (control, prevention, treatment) 60 days after radiation. **(b)** Quantification of mRNA expression for COL-1A1, COL-3A1 and fibronectin in the mouse intestine survived 28 days post-radiation (n=3). **(c)** Quantitation of tongues stained with toluidine blue from normal mice and irradiated mice (control, prevention, treatment) 10 days after radiation. The ulcer area was calculated using Image J software (n=3). **(d)** Histological analysis of the tongues 10 days after radiation. **(e)** Survival curve of mice subjected to abdominal radiation (n=15). Bars represent 200μm (a and d). Data in b and c represent the means ± S.D. (**P < 0.01; student's t-test)

Supplementary Figure 4.

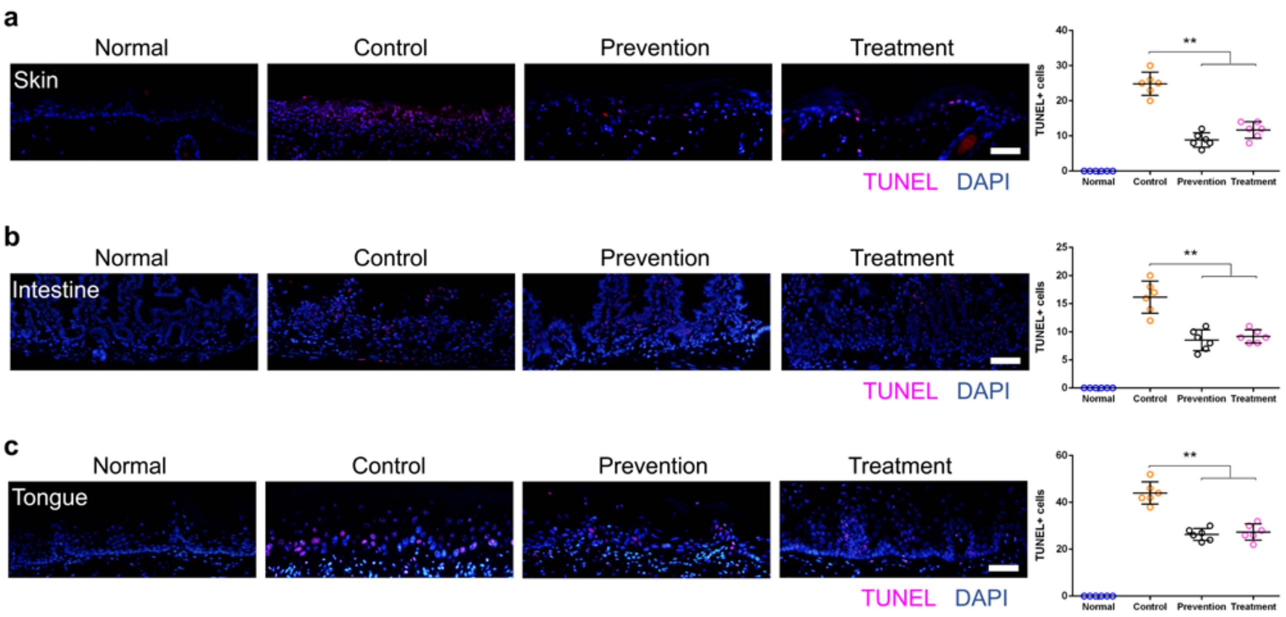

Supplementary Figure 4. Cordycepin prevents the apoptotic cells in radiation ulcer. **(a)** TdT-mediated dUTP nick end labeling (TUNEL) assay in skin sections from normal rats and irradiated rats (control, prevention, treatment) 35 days after irradiation (n=6). **(b)** TUNEL assay in intestine sections from normal mice and irradiated mice (control, prevention, treatment) 4 days after irradiation (n=6). **(c)** TUNEL assay in tongue sections from normal mice and irradiated mice (control, prevention, treatment) 10 days after irradiation (n=6). The TUNEL positive nuclei were calculated using Image J software. Bars represent 50μm (a-c). Data in a to c represent the means ± S.D. (**P < 0.01; student's t-test)

Supplementary Figure 5.

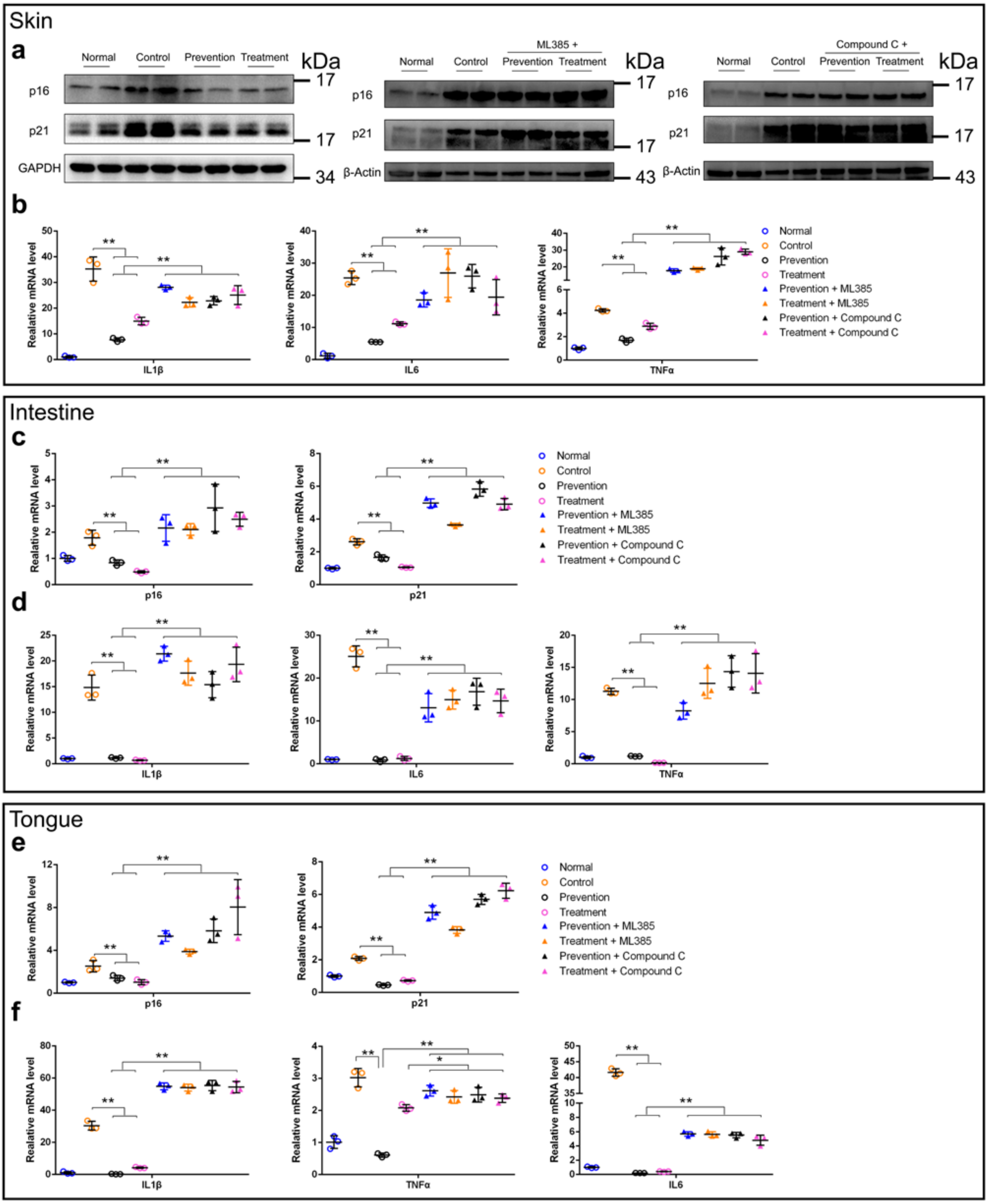

Supplementary Figure 5. Cordycepin prevents cell senescence in vivo. **(a)** Western blot analysis of p16 and p21 levels in skin tissues from normal rats and irradiated rats 35 days

after radiation. **(b)** Quantification of mRNA expression for IL1β, IL6 and TNFα in skin tissues from normal rats and irradiated rats 35 days after radiation. **(c-f)** Quantification of mRNA expression for p16, p21, IL1β, IL6 and TNFα in intestinal and tongue sections from normal mice and irradiated mice 4 and 10 days after irradiation, respectively. Data in b to f represent the means ± S.D. (n=3, *P < 0.05, **P < 0.01; student's t-test)

Supplementary Figure 6.

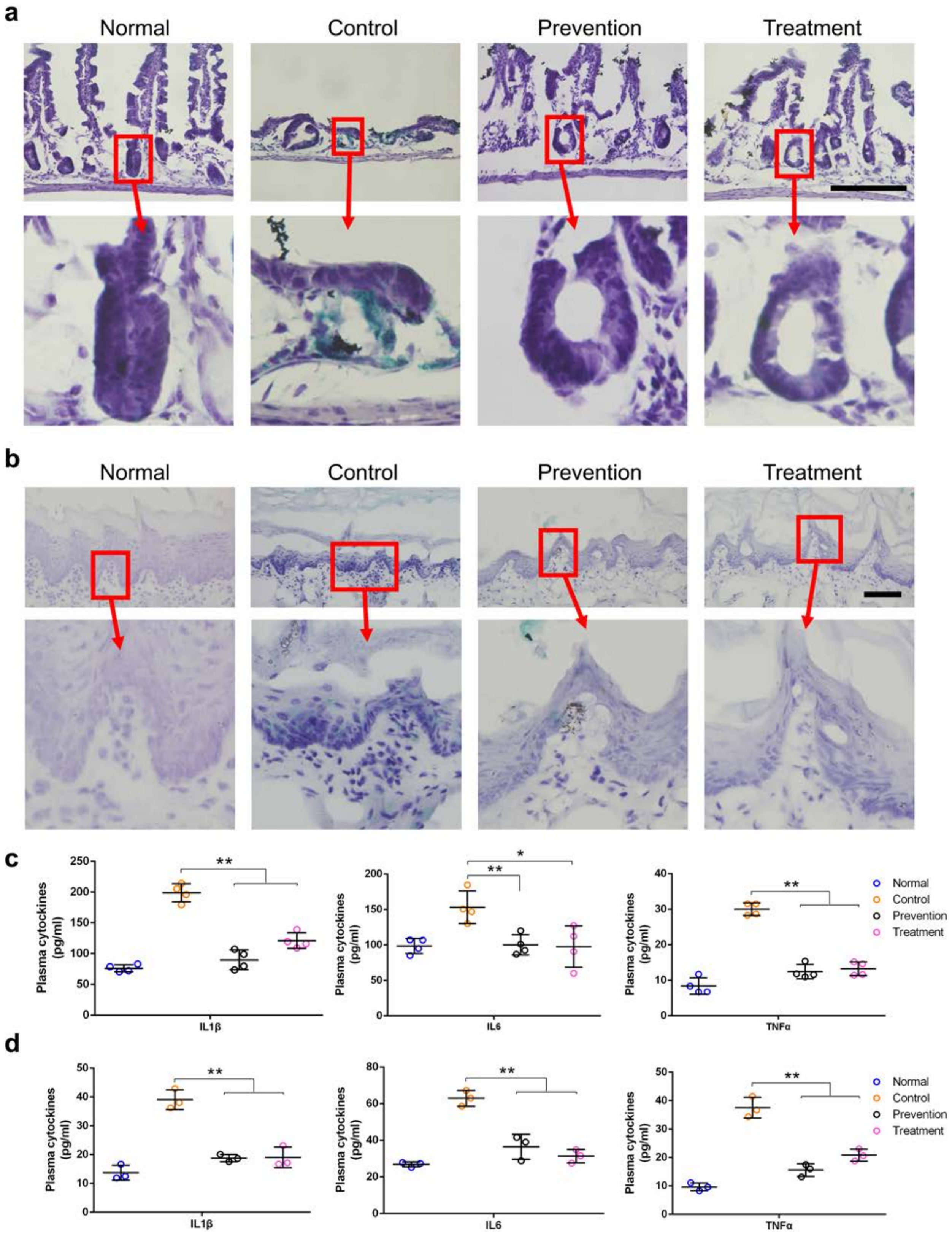

Supplementary Figure 6. Cordycepin reduces SA-β-gal^{+} cells and SASP in vivo. **(a)** Representative SA-β-gal staining in intestine tissues from normal mice and irradiated mice

(control, prevention, treatment) 4 days after radiation. **(b)** Representative SA-β-gal staining in intestine tissues from normal mice and irradiated mice (control, prevention, treatment) 10 days after radiation. **(c-d)** Plasma concentrations of cytokines in the circulating levels of blood IL1β, IL6 and TNFα from rat (c) and mouse (d). Bars represent 100μm(a), 50μm(b). Data in c to d represent the means ± S.D. (n=3, *P < 0.05, **P < 0.01; student's t-test)

Supplementary Figure 7.

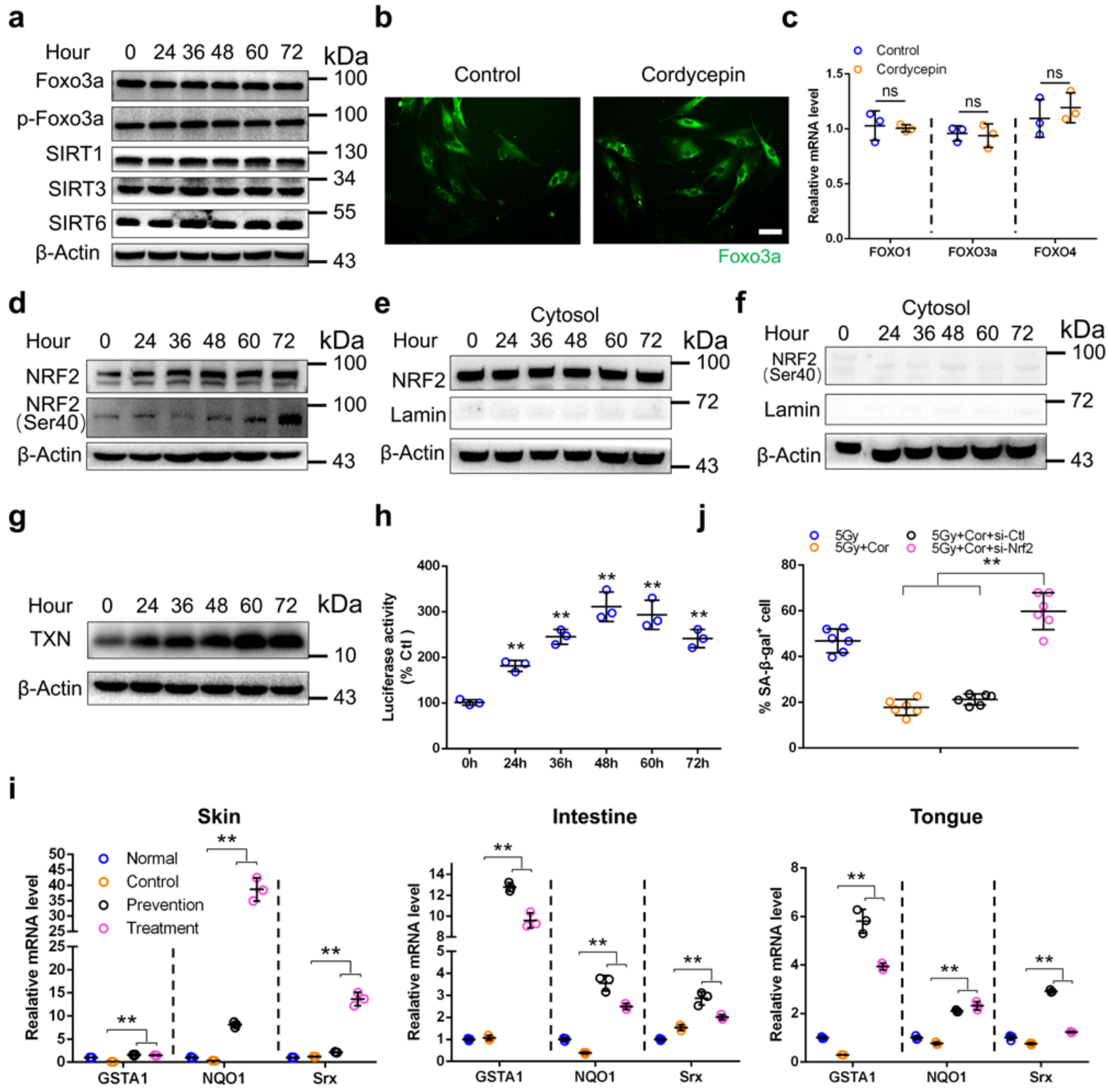

Supplementary Figure 7. Cordycepin prevents senescence which is NRF2-dependent. **(a)** Western blot analysis of Foxo3a, p-Foxo3a, SIRT1, SIRT3, SIRT6 levels from 200μM cordycepin-treated fibroblasts for the indicated times. **(b)** Representative immunofluorescence pictures of Foxo3a in control and cordycepin-treated fibroblasts for 3 days. **(c)** Quantification of mRNA expression for Foxo1, Foxo3a, Foxo4 from cordycepin-treated fibroblasts for 3 days (n=3). **(d)** Western blot analysis of NRF2 and

p-NRF2 levels from 200μM cordycepin-treated HaCaT cells for the indicated times. **(e-f)** NRF2 and p-Nrf2 expression levels in cytosol from cordycepin-treated fibroblasts for the indicated times. **(g)** Western blot analysis of TXN levels from 200μM cordycepin-treated fibroblasts for the indicated times. **(h)** ARE-driven luciferase activity from 200μM cordycepin-treated fibroblasts for the indicated times (n=3). **(i)** Quantification of mRNA expression for GSTA1, NQO1 and Srx in skin, intestinal and tongue sections from normal mice/rats and irradiated mice/rats (control, prevention, treatment) 35, 4 and 10 days after radiation, respectively (n=3). **(j)** Quantification of SA-β-gal 7 days after radiation, fibroblasts were incubated in the absence or presence of 40μM Compound C (C.C) for 4 hours in advance and then were treated with 200μM cordycepin for 3 days (kept with C.C) before irradiation (n=6). Bars represent 50μm (b). Data in c, h, i and j represent the means ± S.D. (**P < 0.01; student's t-test; ns not significant)

Supplementary Figure 8.

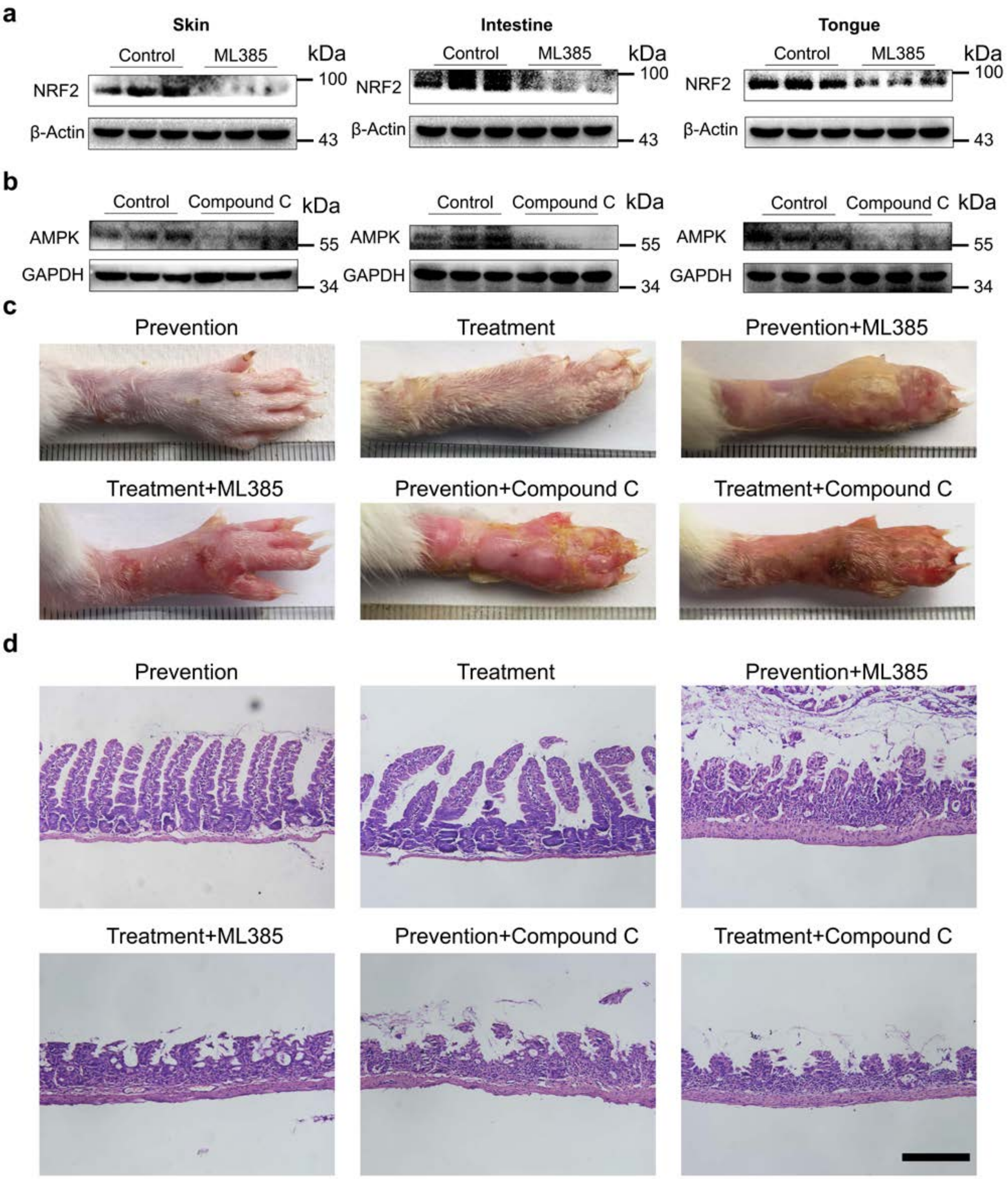

Supplementary Figure 8. AMPK-NRF2 pathway axis involved in cordycepin-mediate anti-ulcer effects. **(a-b)** Mice/Rats were injected intraperitoneally with ML385 or compound C every day for 7 days, the rat skin, mouse intestine and mouse tongue tissues were

harvested for western blot analysis of NRF2 or AMPK levels. **(c)** Images of hind limb from normal rats and irradiated rats on day 35. **(d)** Histological analysis of intestinal sections from normal mice and irradiated mice 4 days after radiation. Bars represent 200μm (d).

Supplementary Figure 9.

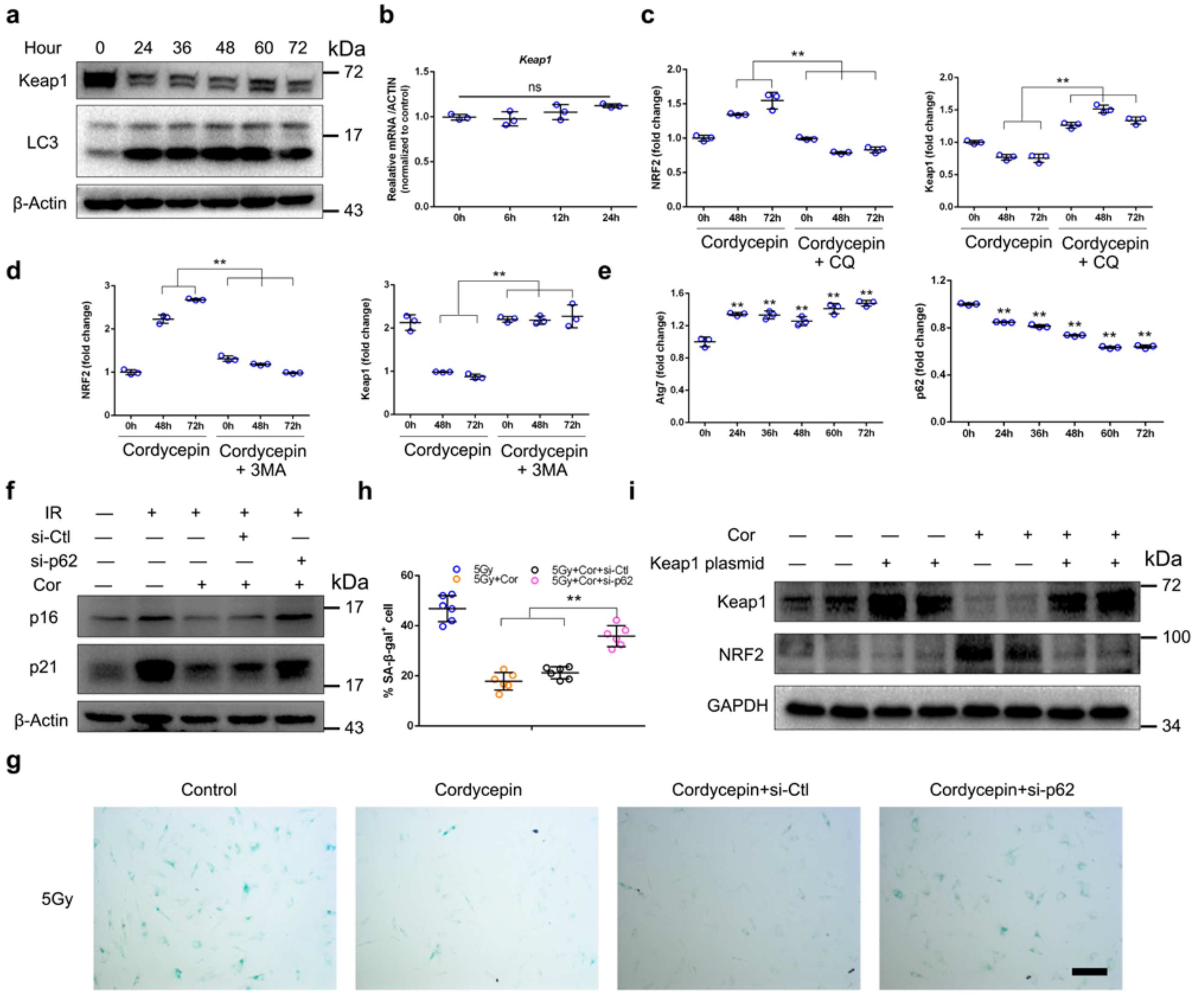

Supplementary Figure 9. Cordycepin activates NRF2 by promoting autophagic degradation of Keap1. **(a)** Western blot analysis of KEAP1 and LC3 levels from 200μM cordycepin-treated HaCaT cells for the indicated times. **(b)** Quantification of mRNA expression for Keap1 from cordycepin-treated fibroblasts for the indicated times. **(c-e)** Densitometry quantified data of NRF2, Keap1, Atg7 and p62 to GAPDH or β-Actin expression ratios, represented as a fold change to non-stimulated cells. **(f)** Western blot analysis of p16 and p21 levels in irradiated control or cordycepin treated (3 days) fibroblasts 3 days following knockdown of p62 7 days after radiation. **(g-h)** Staining and quantification

for senescence-associated β-galactosidase (SA-β-gal) 7 days after radiation in fibroblasts following knockdown of p62. **(i)** Fibroblasts were transfected with a Keap1 plasmid, and then treated with vehicle control or 200μM cordycepin for 72 hours, total proteins were harvested for detection of Keap1 and NRF2. Bars represent 250μm (g). Data in b to e, h represent the means ± S.D. ($n=3$, $^{**}P < 0.01$; student's t-test; ns not significant)

Supplementary Figure 10.

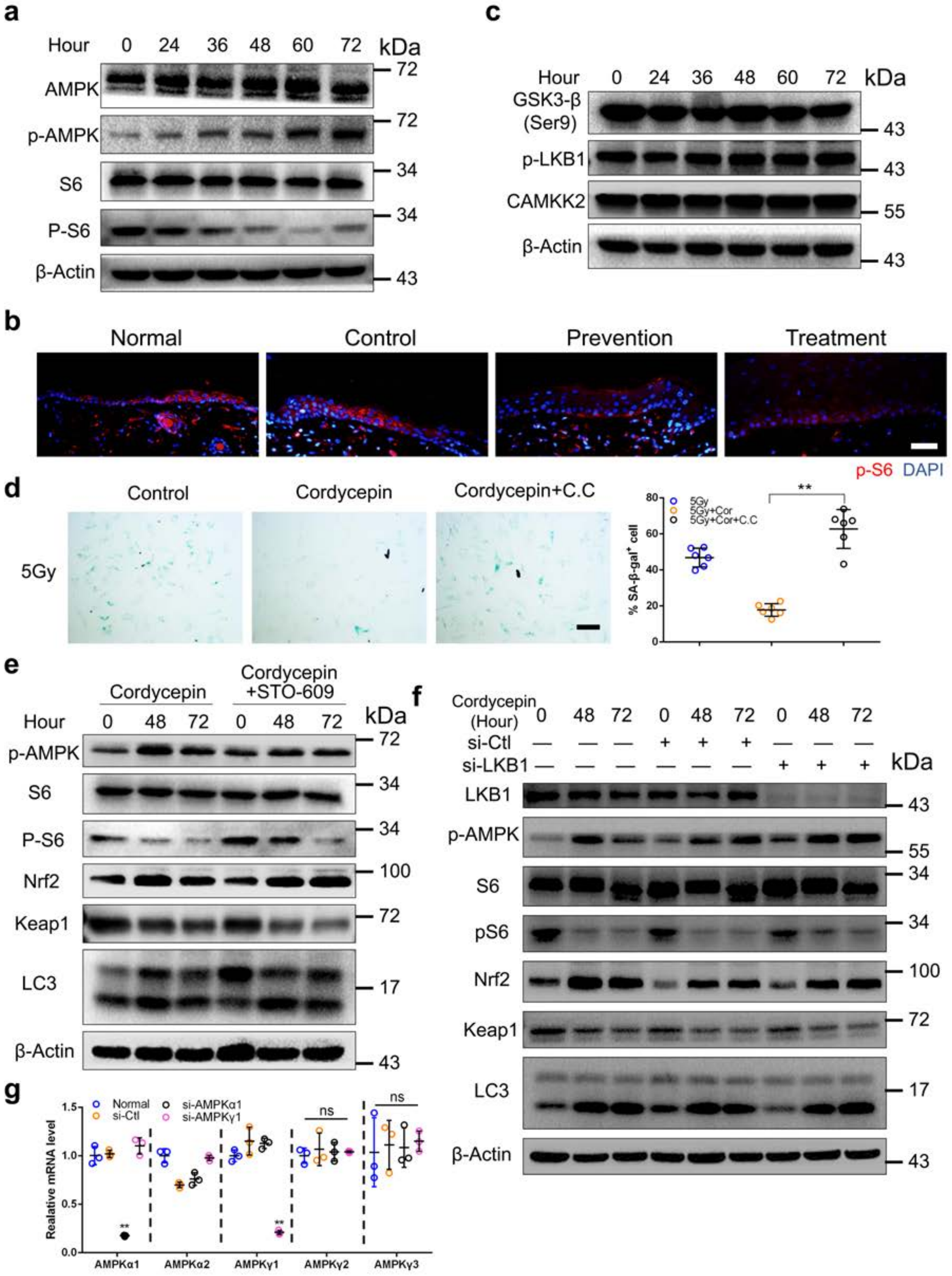

Supplementary Figure 10. Cordycepin interacts with α1 and γ1 subunit of AMPK. **(a)** Western blot analysis of AMPK, p-AMPK, S6, and p-S6 levels from 200μM

cordycepin-treated HaCaT cells for the indicated times. **(b)** Representative immunofluorescence pictures of p-S6 of the skin tissues from normal animals and irradiated animals (control, prevention, treatment) 35 days after radiation dose. **(c)** Western blot analysis of GSK-3β(Ser9), p-LKB1and CAMKK2 levels from 200μM cordycepin-treated fibroblasts for the indicated times. **(d)** Staining and quantification of SA-β-gal 7 days after radiation, fibroblasts were incubated in the absence or presence of 40μM Compound C (C.C) for 4 hours in advance and then were treated with 200μM cordycepin for 3 days (kept with C.C) before radiation (n=6). **(e)** Fibroblasts were incubated in the absence or presence of 10 μg/ml STO-609 for 4 hours in advance and then were treated with 200μM cordycepin for the indicated times (kept with STO-609), and total proteins were harvested for detection of p-AMPK, S6, p-S6, NRF2, KEAP1 and LC3 by Western blot. **(f)** Fibroblasts were transfected with control siRNA (si-Ctl), siRNA against LKB1 (si-LKB1) and then were treated with 200μM cordycepin for 3 days, and total proteins were harvested for detection of LKB1, p-AMPK, S6, p-S6, NRF2, KEAP1 and LC3 by Western blot. **(g)** Quantification of mRNA expression for AMPKα1/2 and AMPKγ1/2/3 (n=3). Bars represent 100(b), 250μm (d). Data in d and g represent the means ± S.D. (**$P < 0.01$; student's t-test; ns not significant)

Supplementary Figure 11.

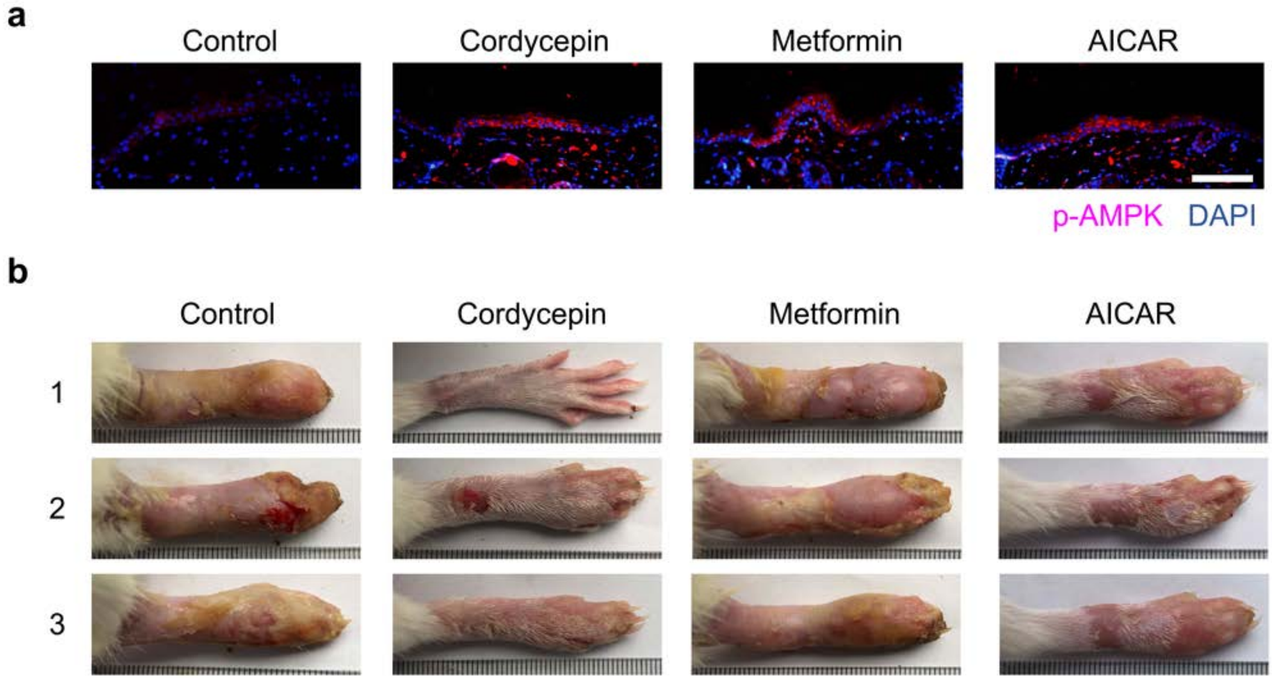

Supplementary Figure 11. Activation of AMPK may represent new therapeutic targets for mitigating radiation ulcer. **(a)** Rats were injected intraperitoneally with Cordycepin, Metformin or AICAR every day for 7 days, the rat skin were harvested for immunofluorescence of γ-H2AX. **(b)** Images of hind limb from normal rats and irradiated rats on day 20. Bars represent 50μm (a).

Supplementary Figure 12.

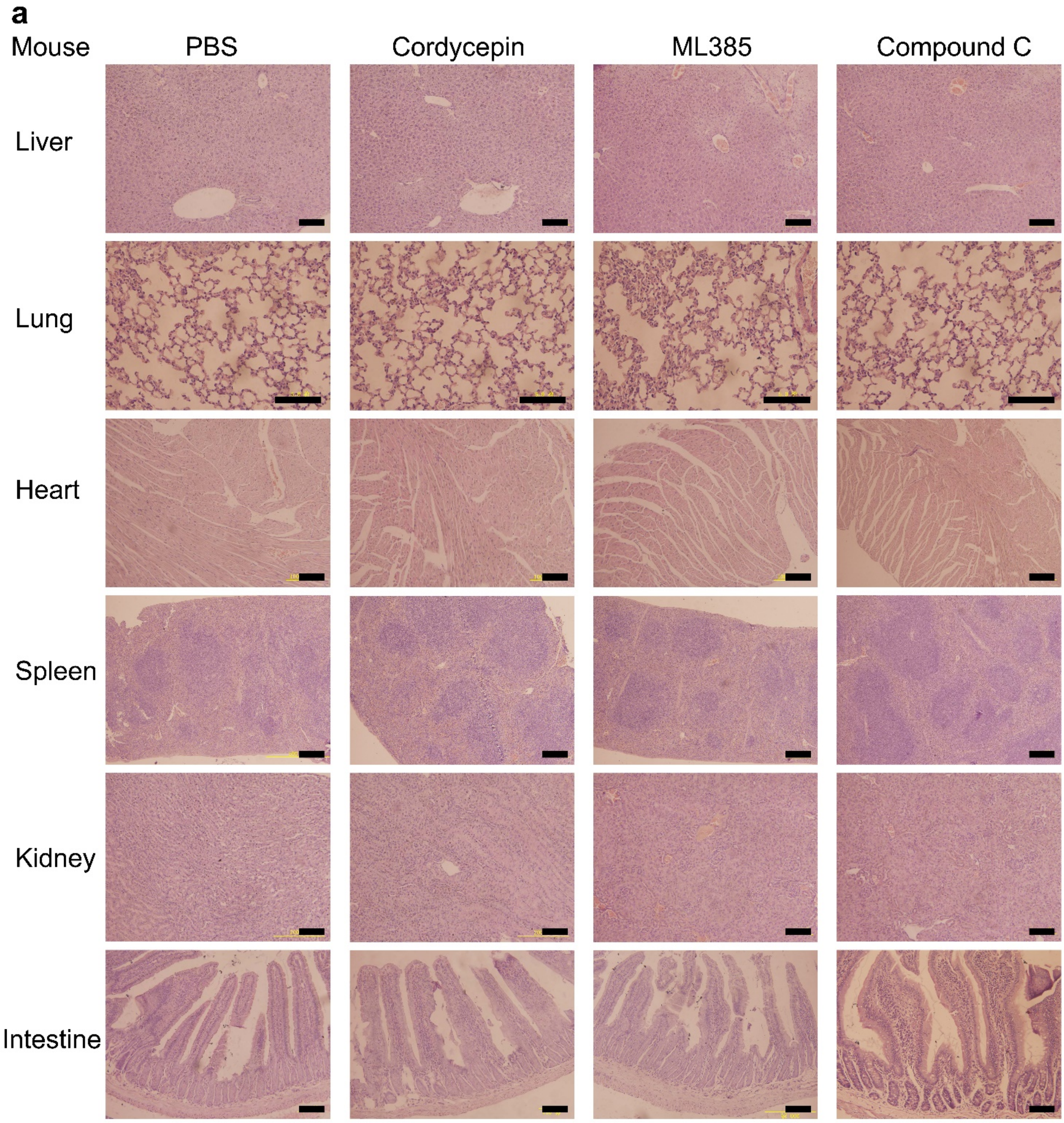

b

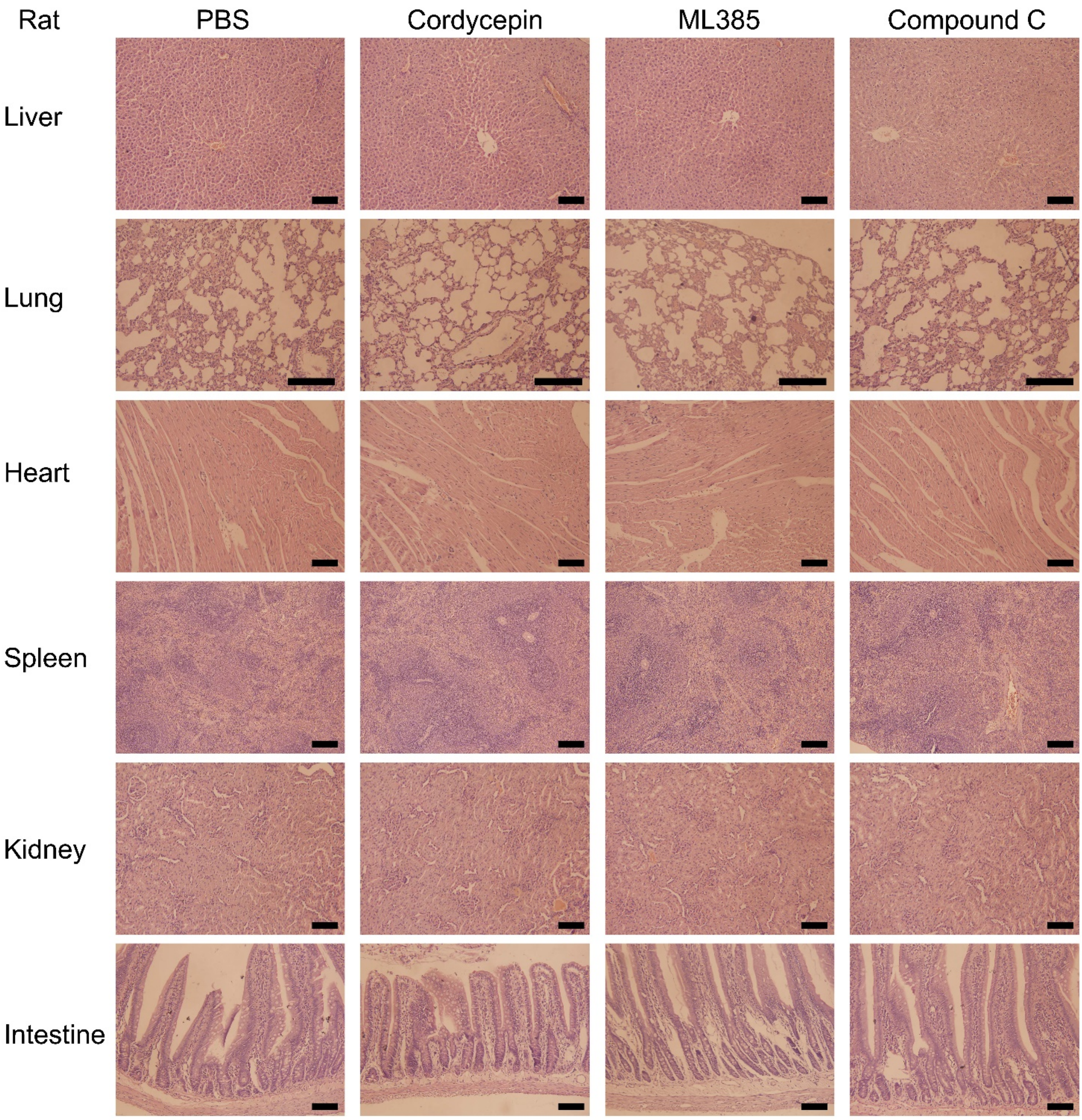

Supplementary Figure 12. Cordycepin, ML385 and compound C could not damage the host vital organs of mice/rats. **(a-b)** Mice(a)/Rats(b) were injected intraperitoneally with vehicle control or cordycepin (60mg/kg), ML385 (30mg/kg), compound C (20mg/kg) every day for 7 days. Lung, liver, spleen, heart, kidney and intestine were collected for HE staining. Bars represent 100μm (liver, heart, kidney, intestine), 200μm (spleen), 50μm (lung).

Supplementary Figure 13.

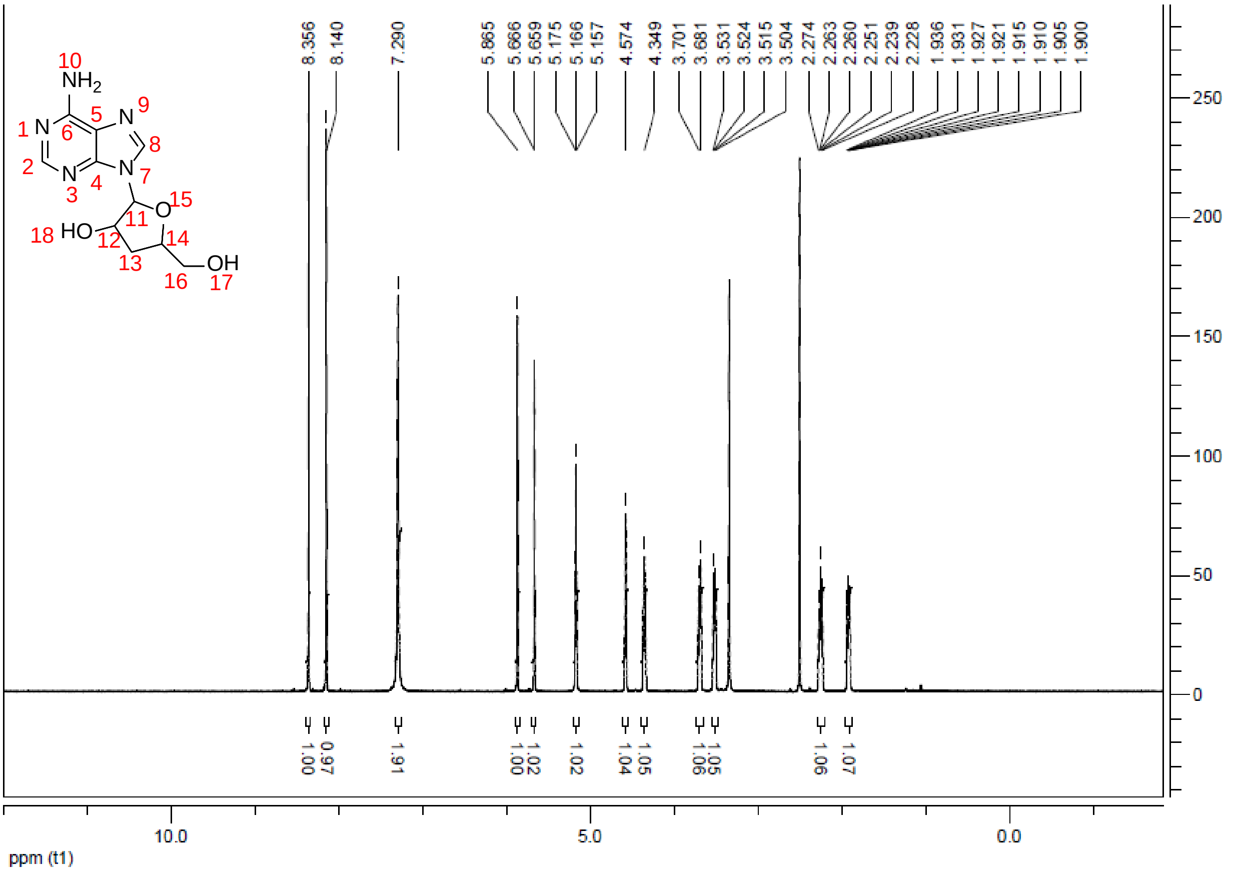

Supplementary Figure 13. ^{1}H NMR of the isolated cordycepin. ^{1}H NMR (600 MHz, DMSO-d_6) δ 8.35(s, 1H, C_2H), 8.12(s, 1H, C_8H), 7.29(s, 2H, NH_2), 5.86(s, 1H, C_{11}H), 5.64(d, J=4.2Hz, 1H, O_{18}H), 5.16(t, J=5.4Hz, 1H, O_{17}H), 4.57(s, 1H, C_{12}H), 4.34 (s, 1H, C_{14}H), 3.69(d, J=12.0Hz, 1H, C_{16}H), 3.53-3.50(m, 1H, C_{16}H), 2.27-2.22(m, 1H, C_{13}H), 1.93-1.90(m, 1H, C_{13}H).

Supplementary Figure 14.

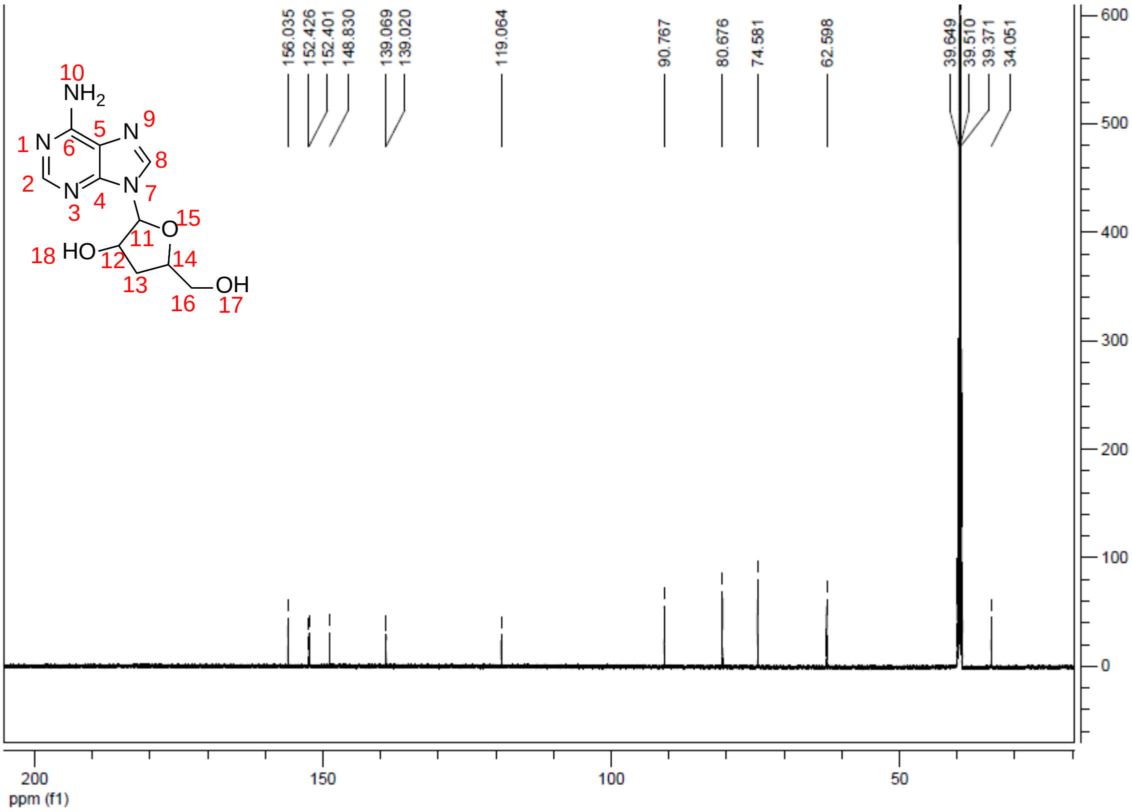

Supplementary Figure 14. [1]C NMR of the isolated cordycepin. ^{13}C NMR (151 MHz, DMSO-d_6) δ 156.03, 152.42, 152.40, 148.83, 139.06, 139.02, 119.06, 90.76, 80.67, 74.58, 62.59, 34.05.

Supplementary Figure 15

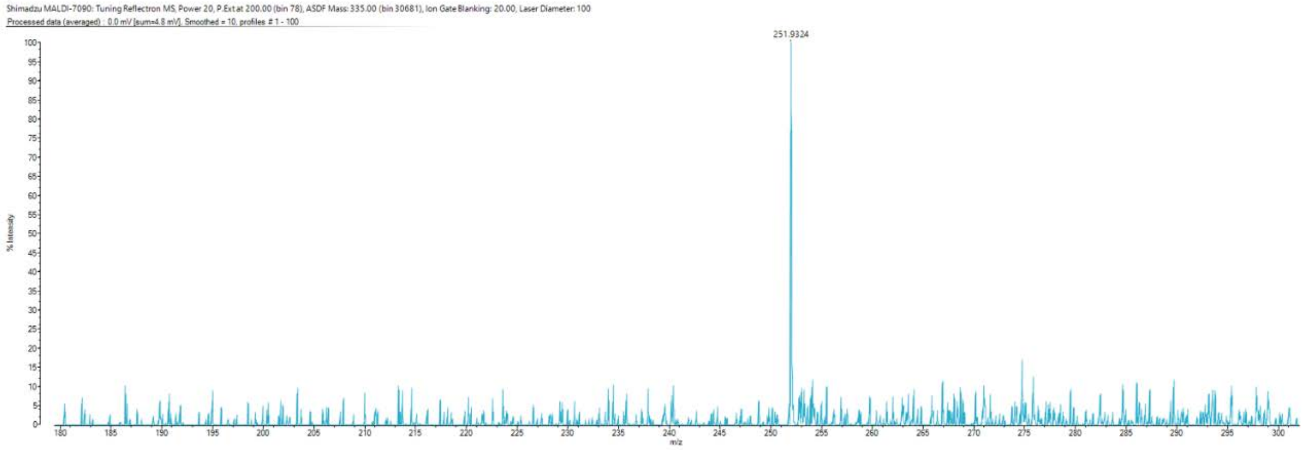

Supplementary Figure 15 | MOLDI-TOF MS of the isolated cordycepin. MS (m/z): calcd for $C_{10}H_{13}N_5O_3$ [M + H]$^+$: 252.1018, found: 251.9324.

Supplementary Figure 16.

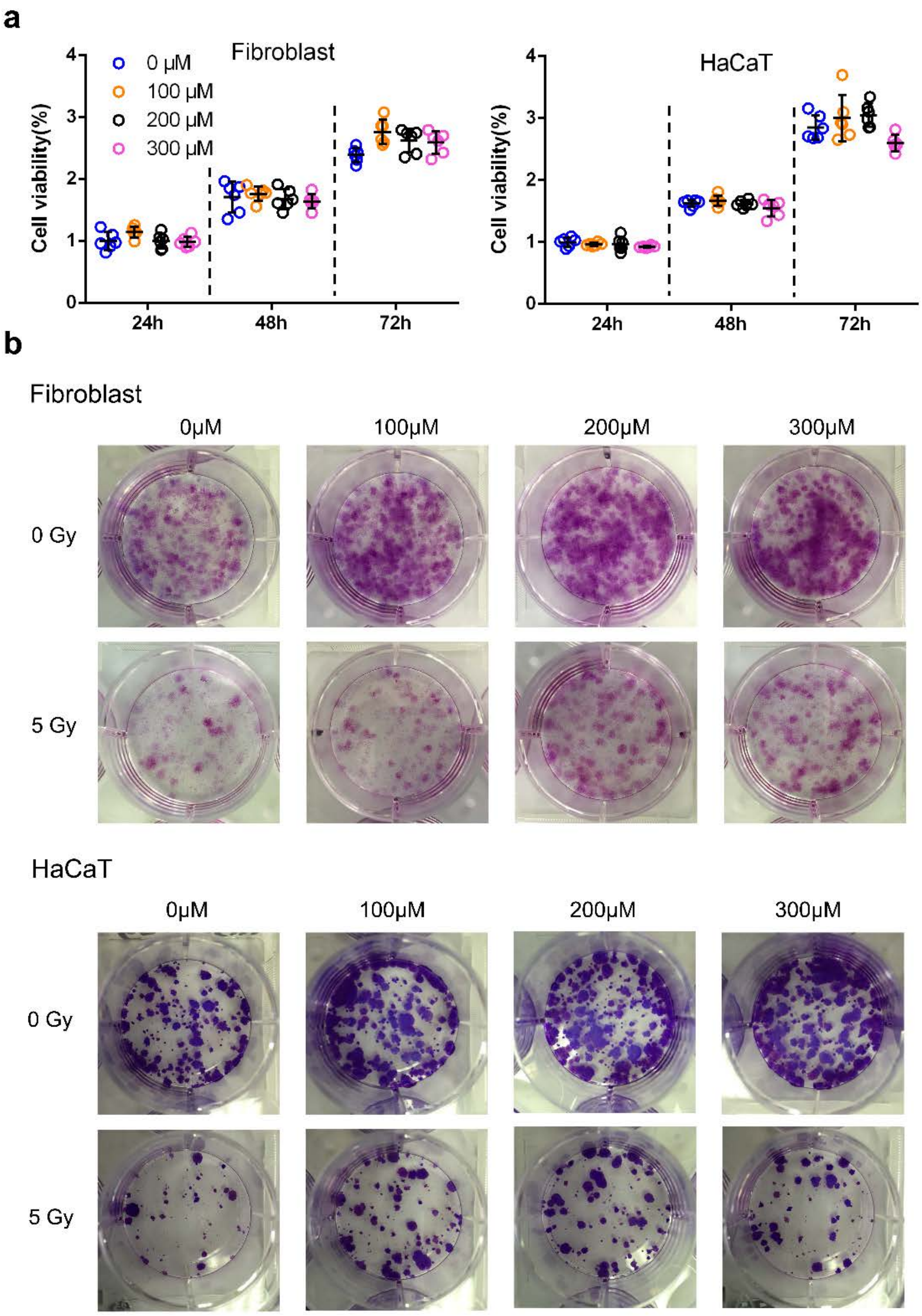

Supplementary Figure 16. The effect of cordycepin on the proliferation and colonies of skin fibroblasts and HaCaT cells. **(a)** Representative proliferation of fibroblasts pretreated with different concentration of cordycepin for indicated times (n=6). **(b)** Representative images of colonies of fibroblasts/HaCaT cells pretreated with different concentration of cordycepin for 72 hours before radiation.

Supplementary Figure 17.

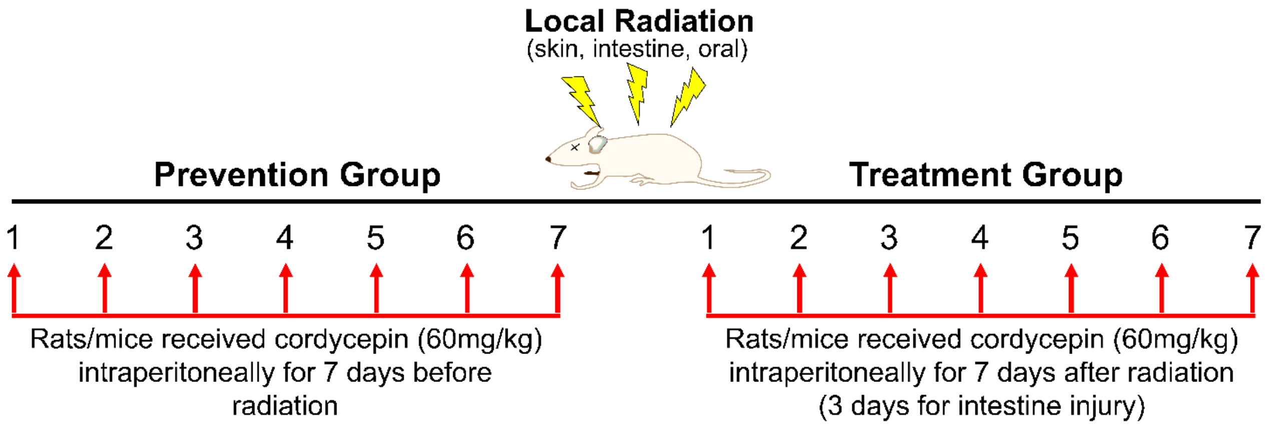

Supplementary Figure 17. The schematic of the animal experimental strategy.

Supplementary Figure 18.

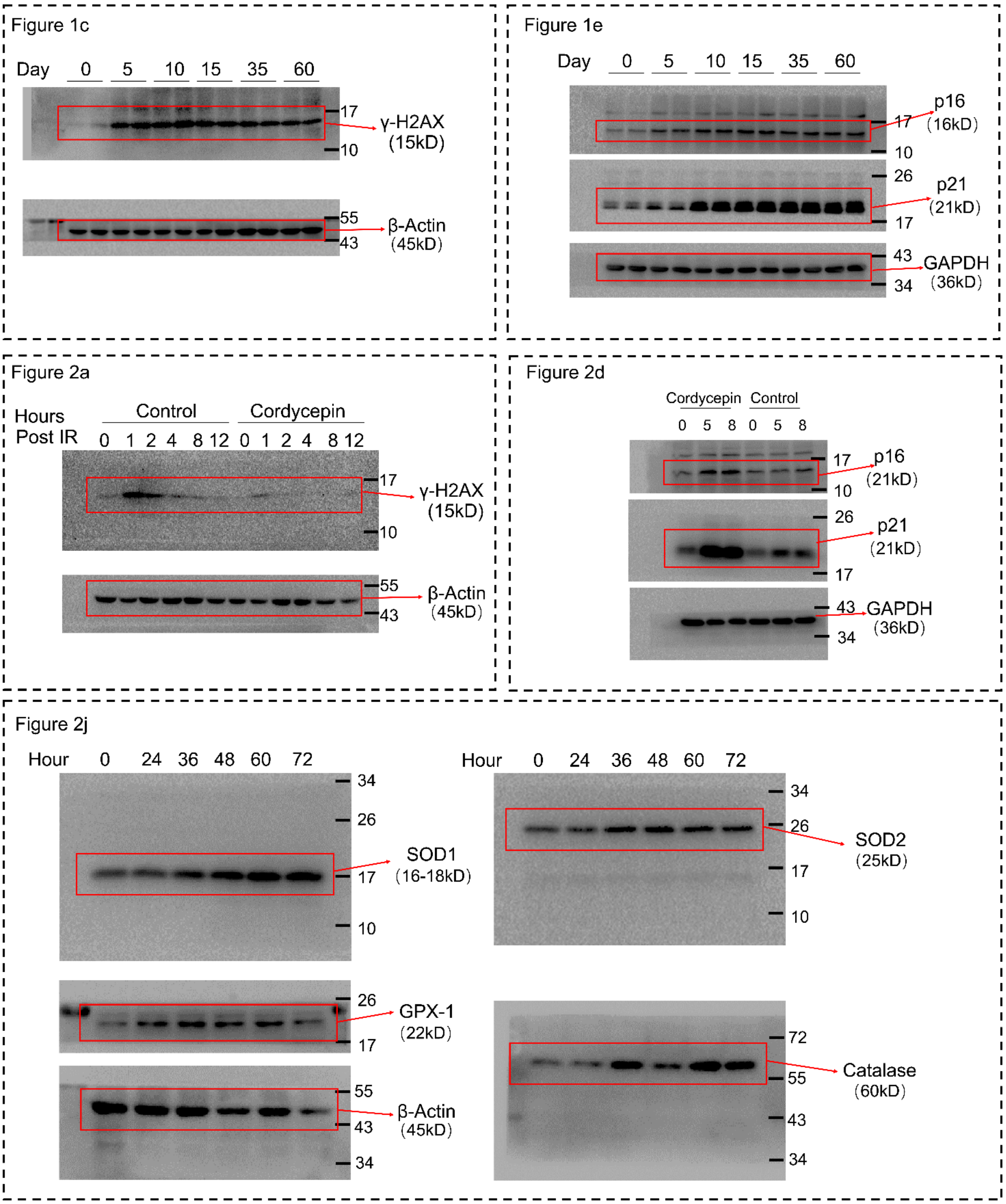

Figure 6a

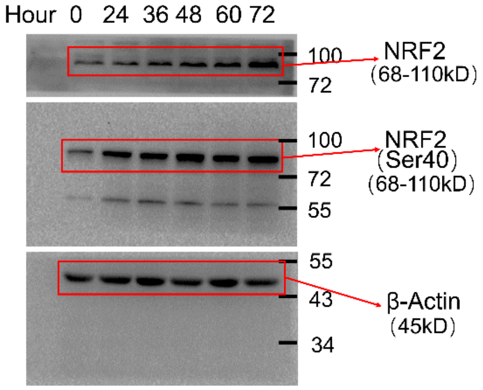

Figure 6b

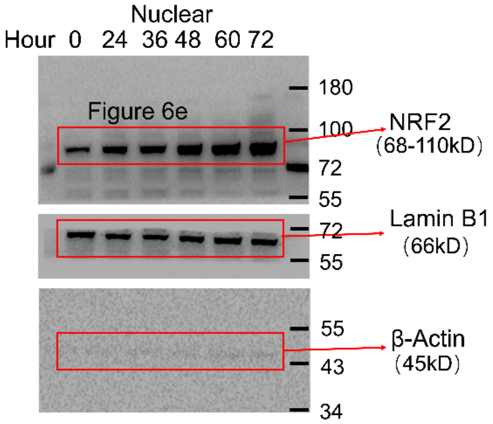

Figure 6e

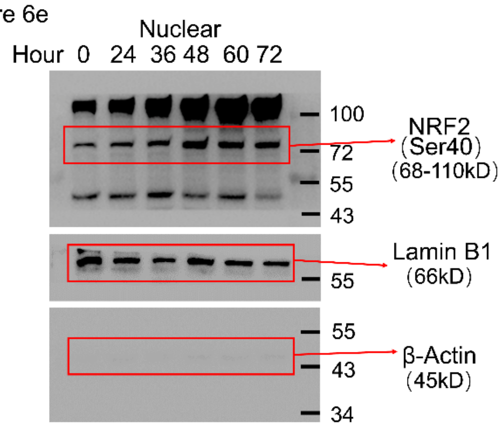

Figure 6h

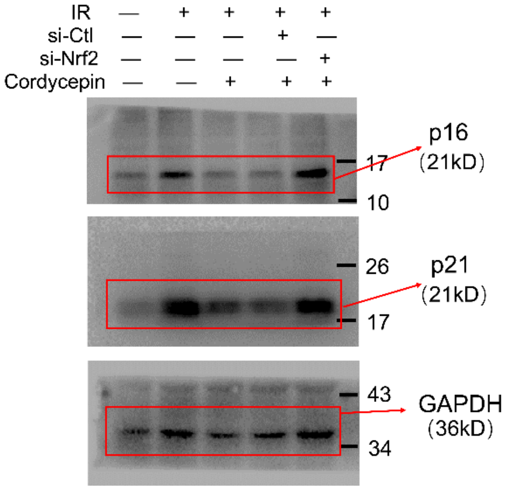

Figure 6g

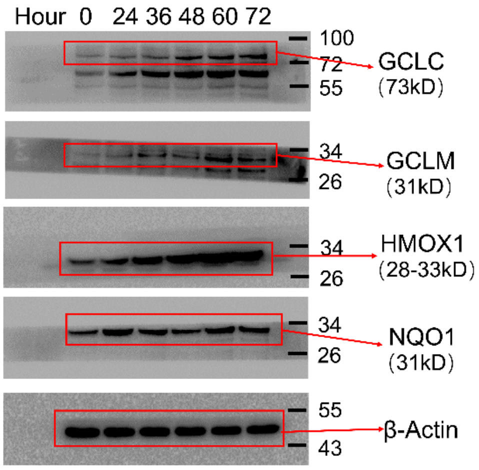

Figure 7b

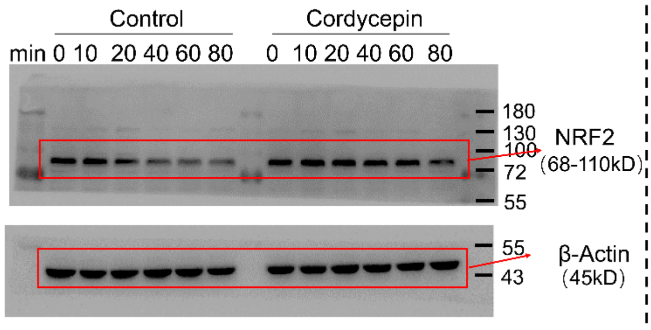

Figure 7c

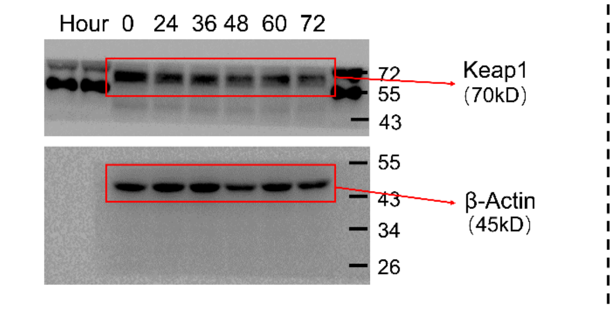

Figure 7e

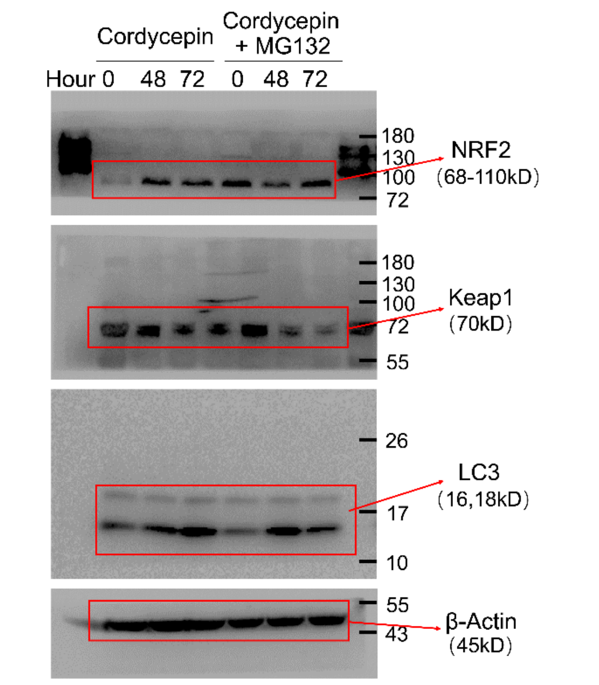

Figure 7f

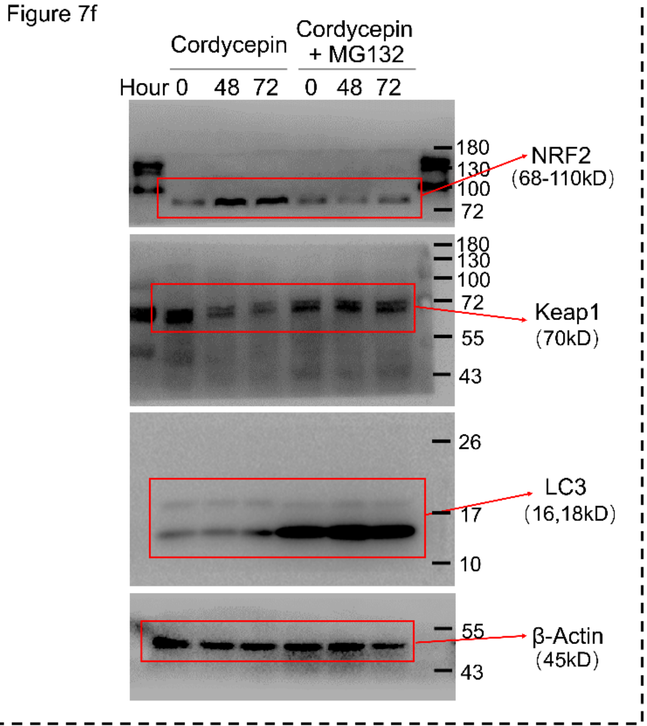

Figure 7g

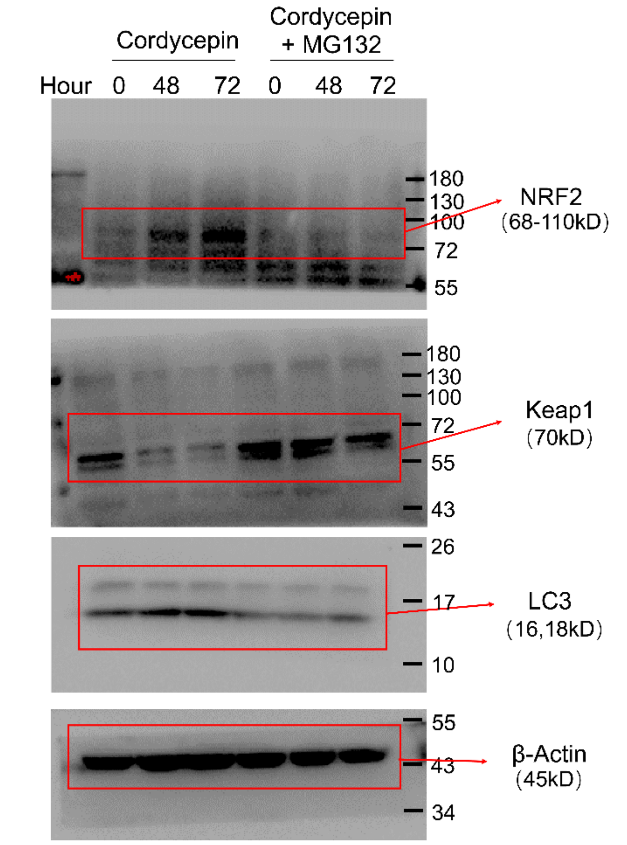

Figure 7h

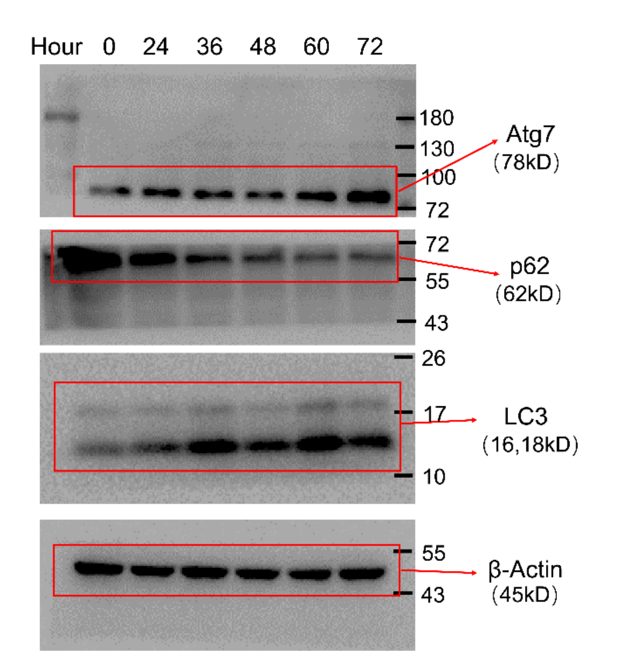

Figure 7j

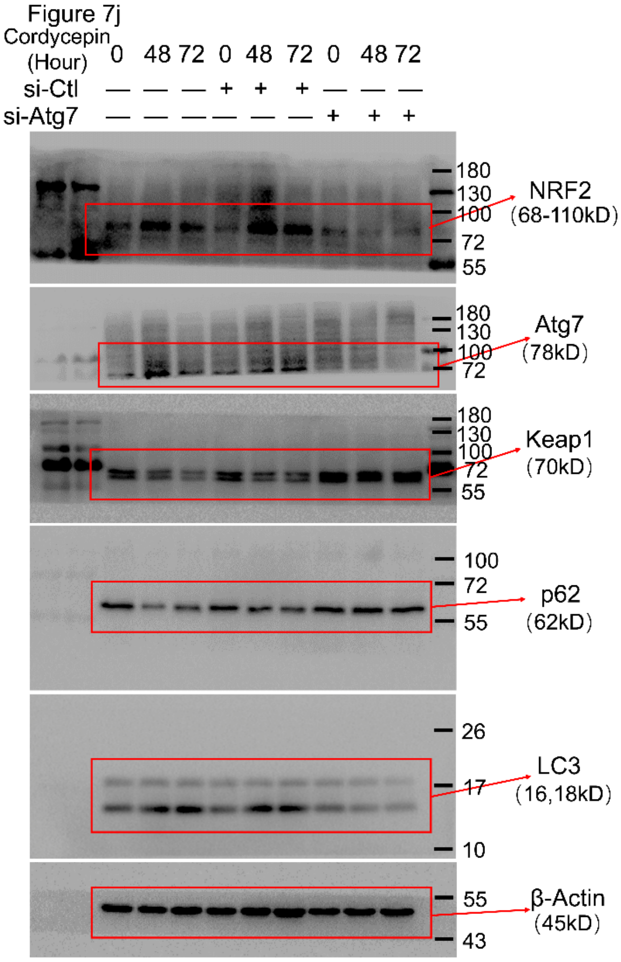

Figure 7k

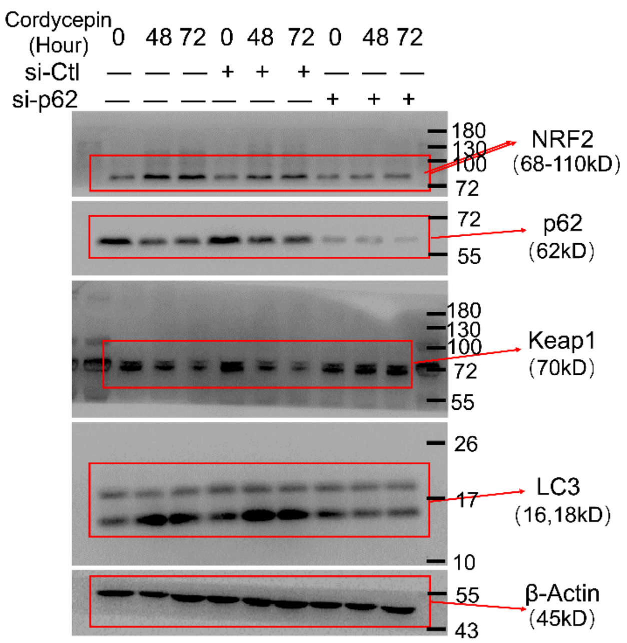

Figure 8a

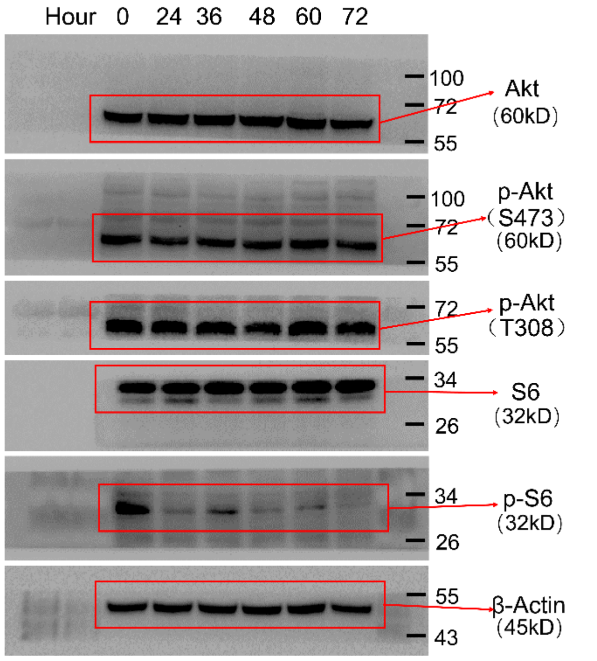

Figure 8c

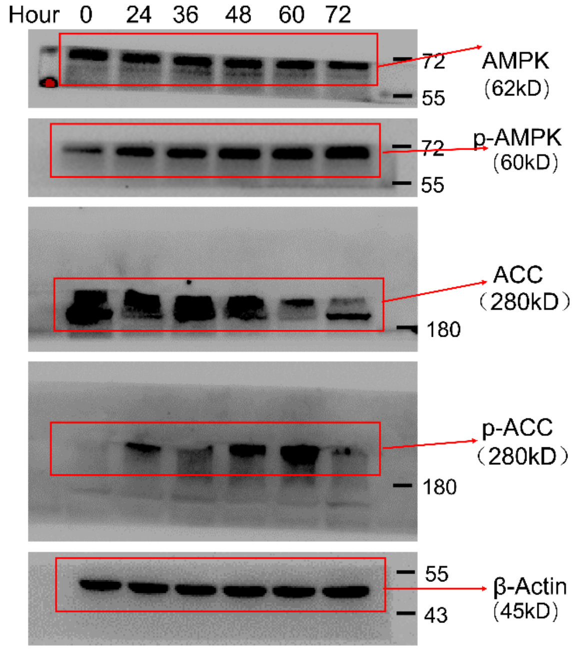

Figure 8d

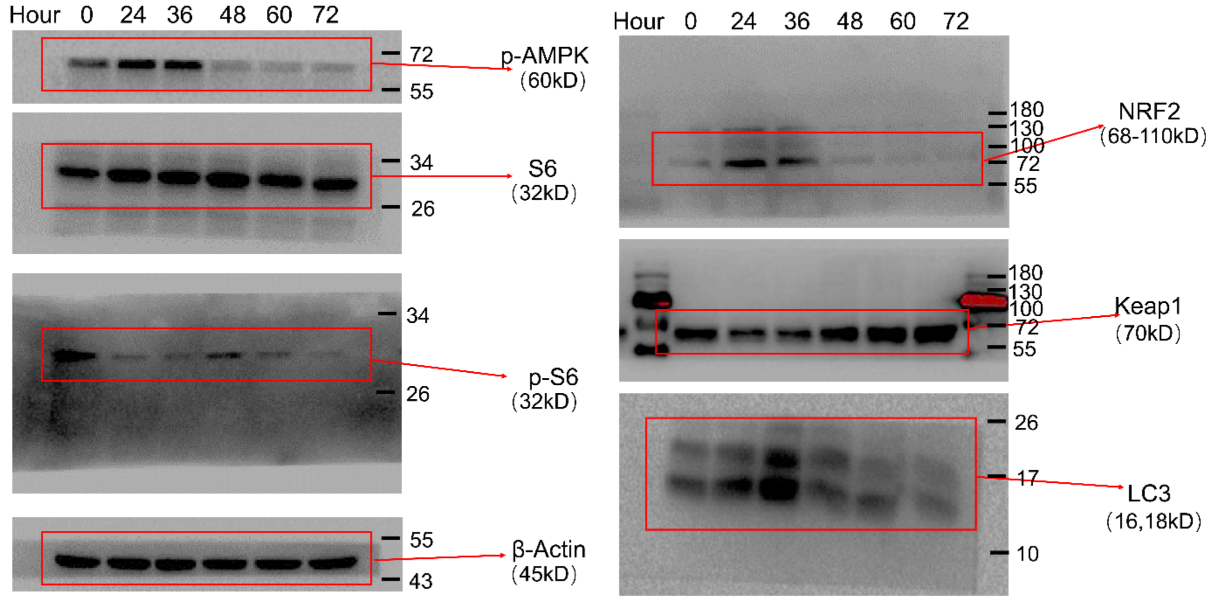

Figure 8g

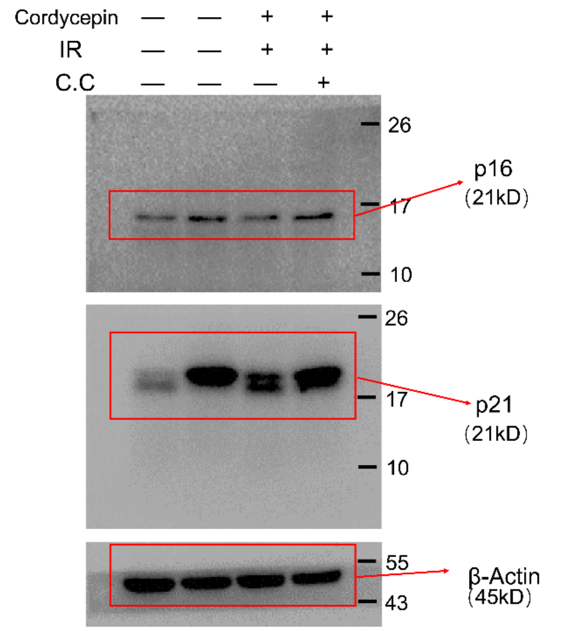

Figure 8j

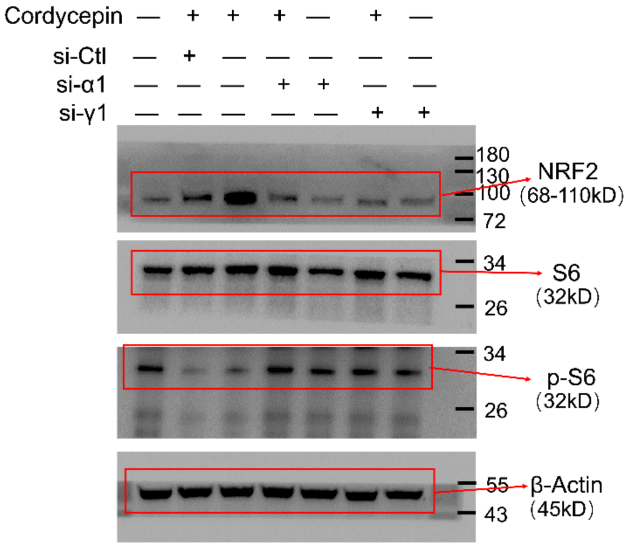

Supplementary figure 1c

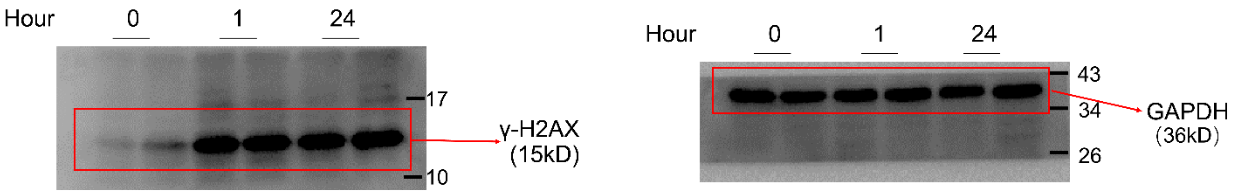

Supplementary figure 2a

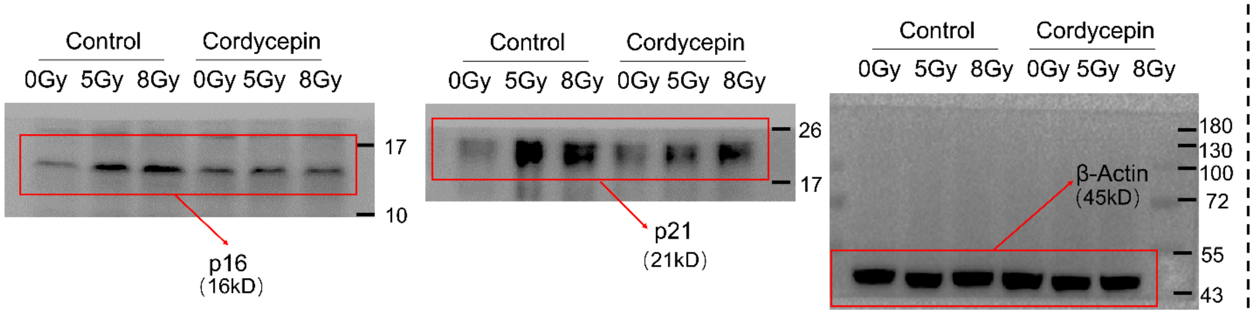

Supplementary figure 5a

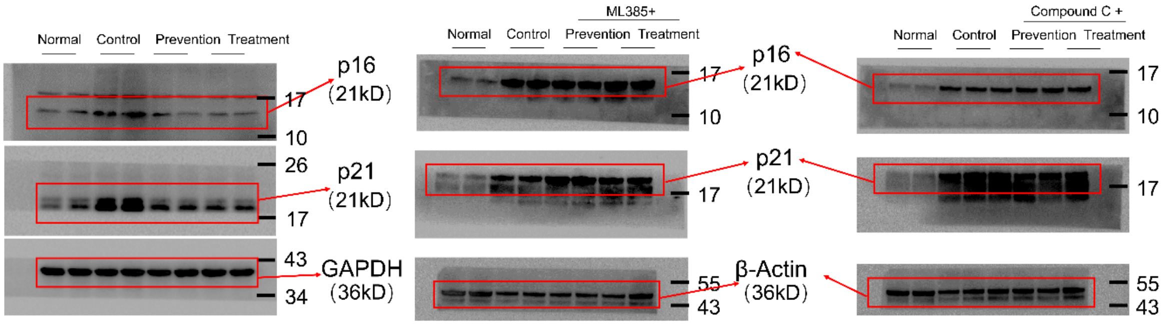

Supplementary figure 7a

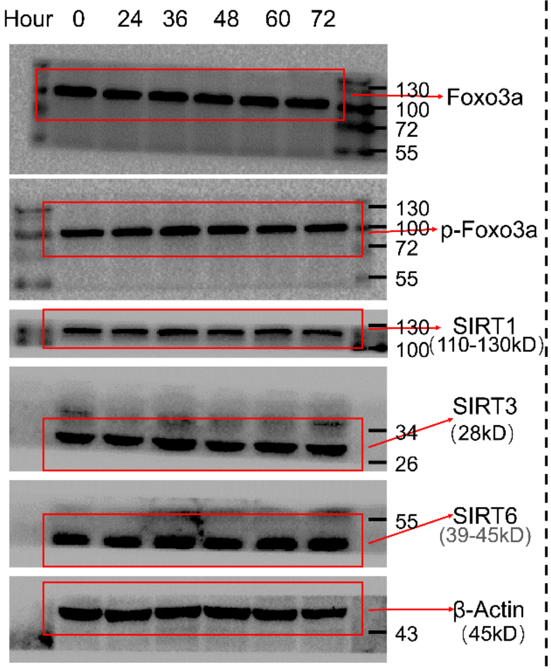

Supplementary figure 7d

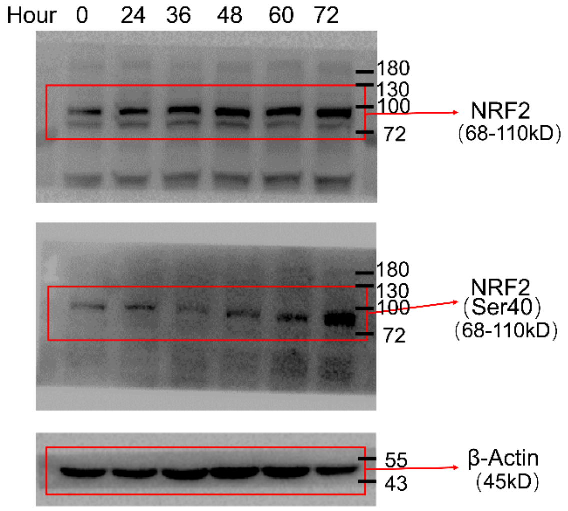

Supplementary figure 7e

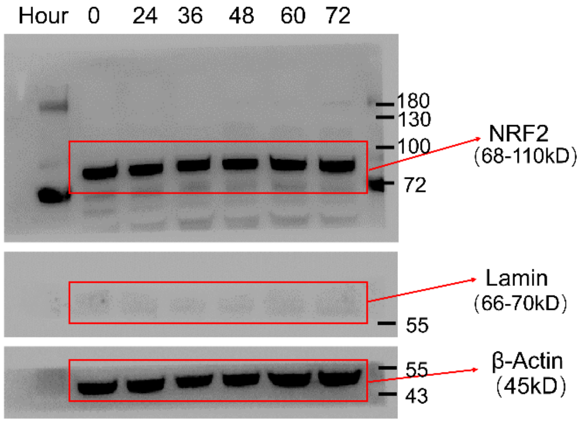

Supplementary figure 7f

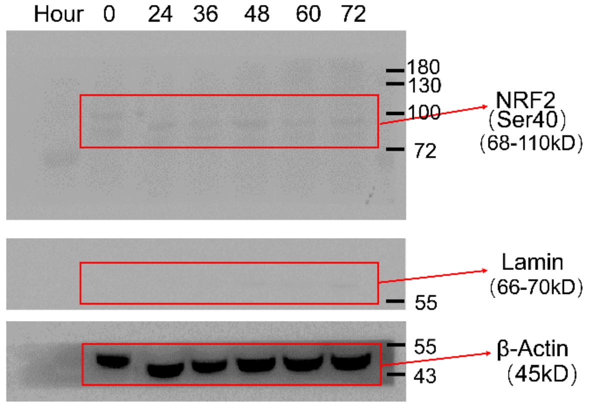

Supplementary figure 7g

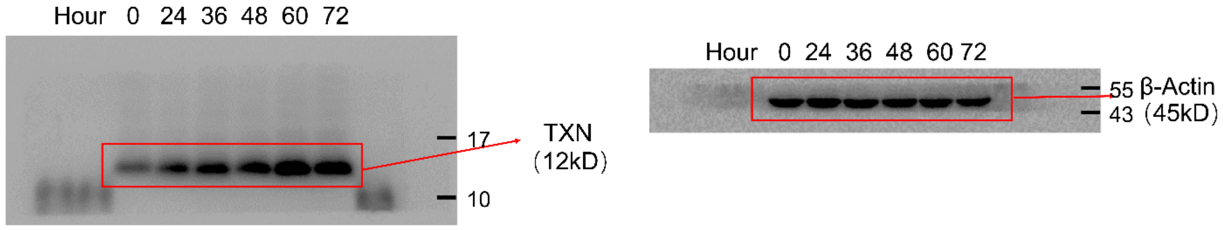

Supplementary figure 8a

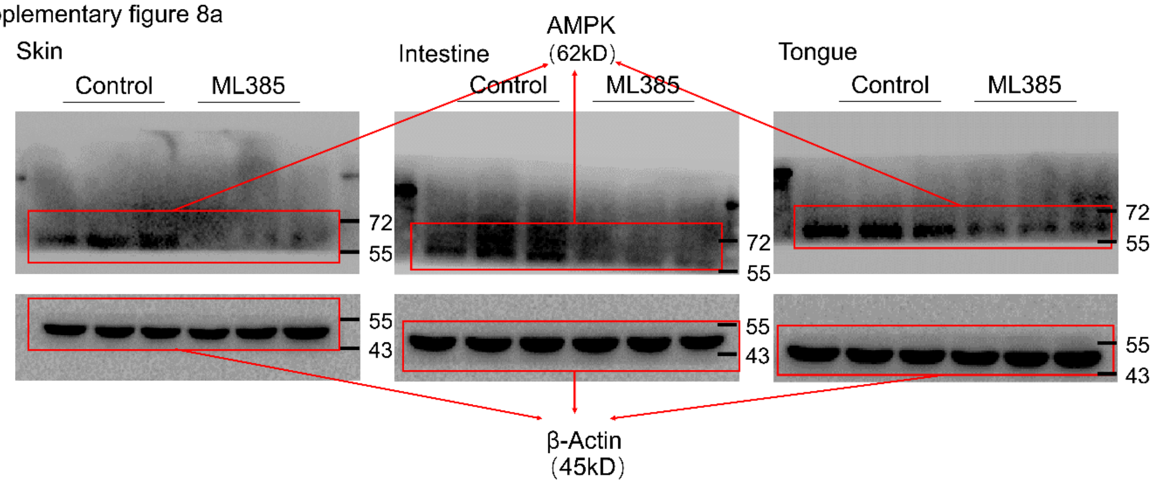

Supplementary figure 8b

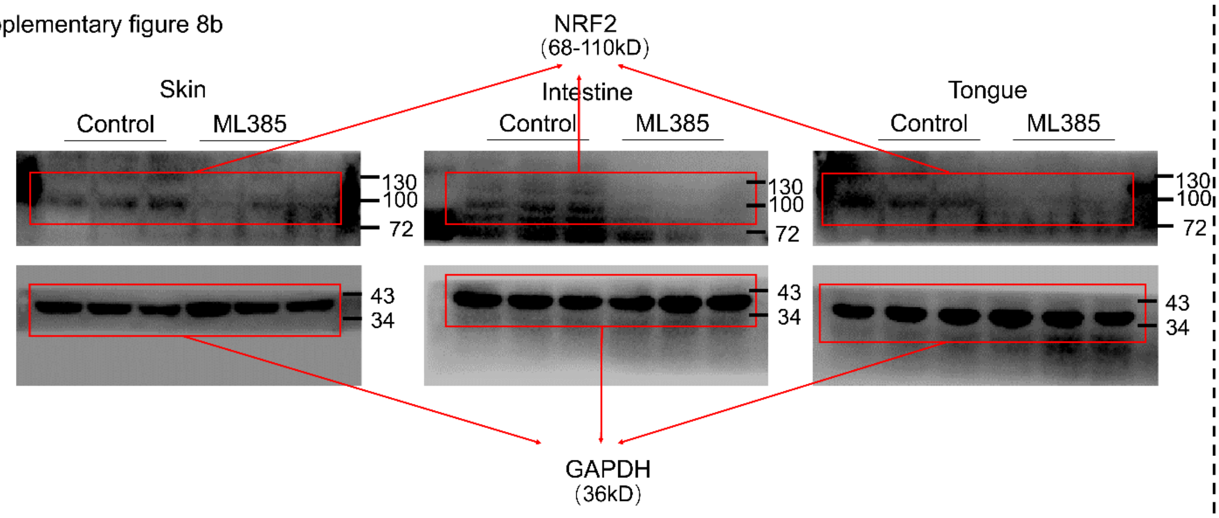

Supplementary figure 9a

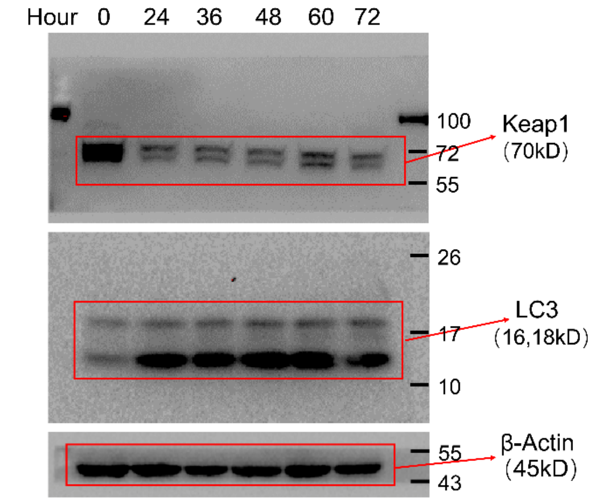

Supplementary figure 9f

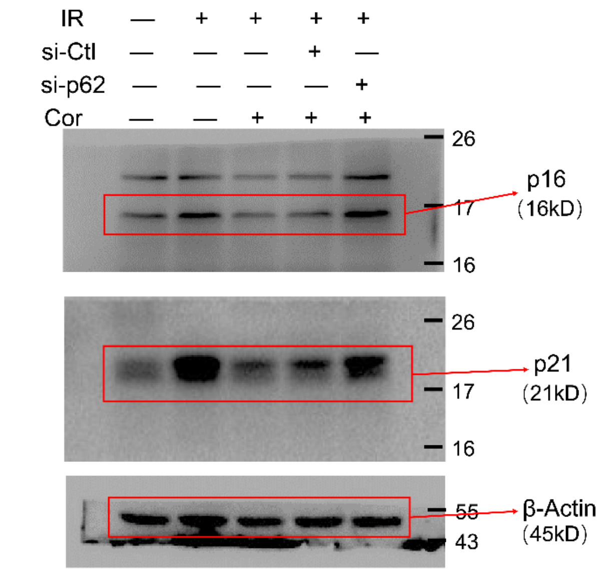

Supplementary figure 9i

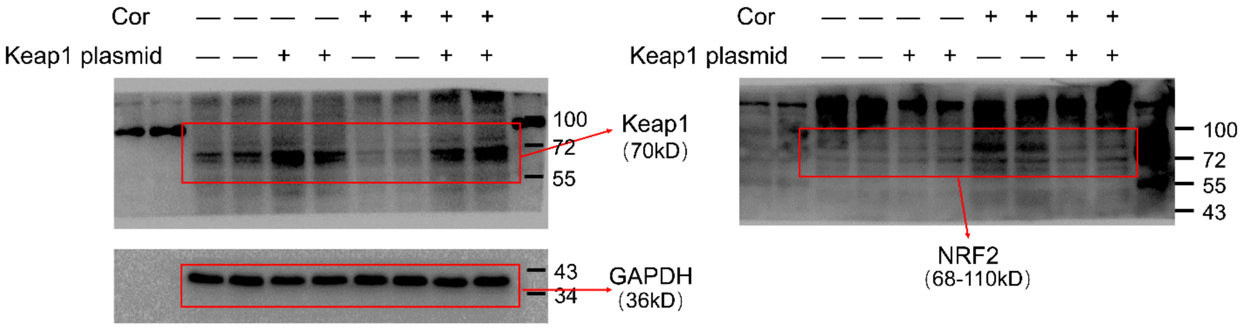

Supplementary figure 10a

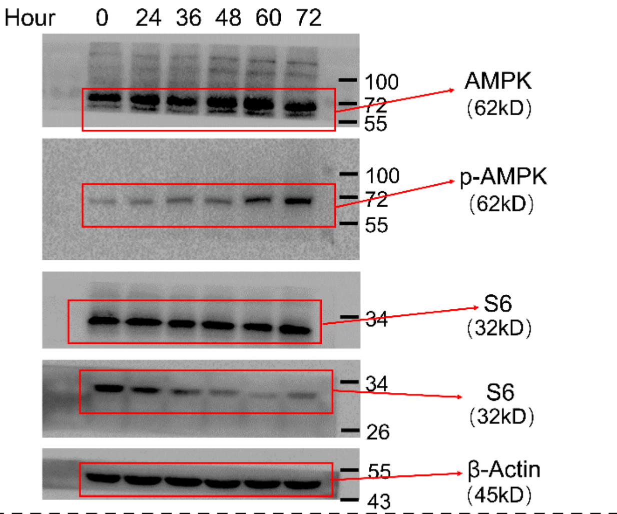

Supplementary figure 10c

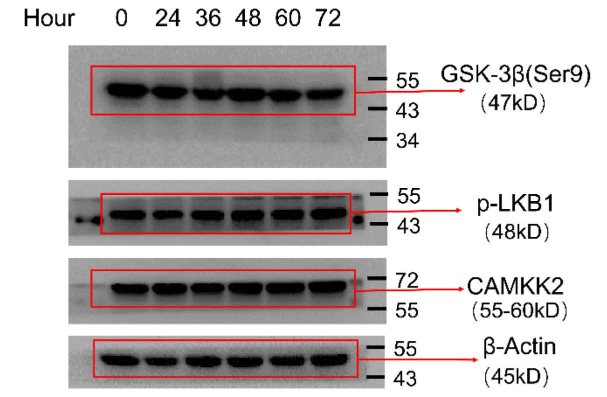

Supplementary figure 10e

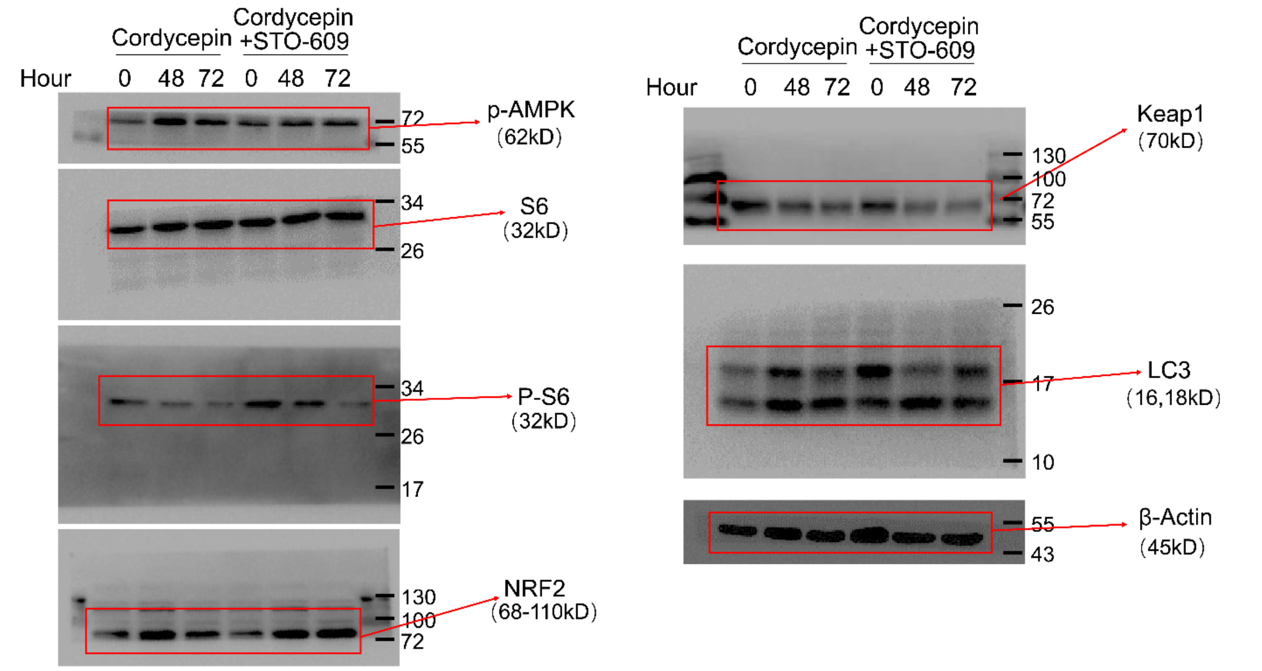

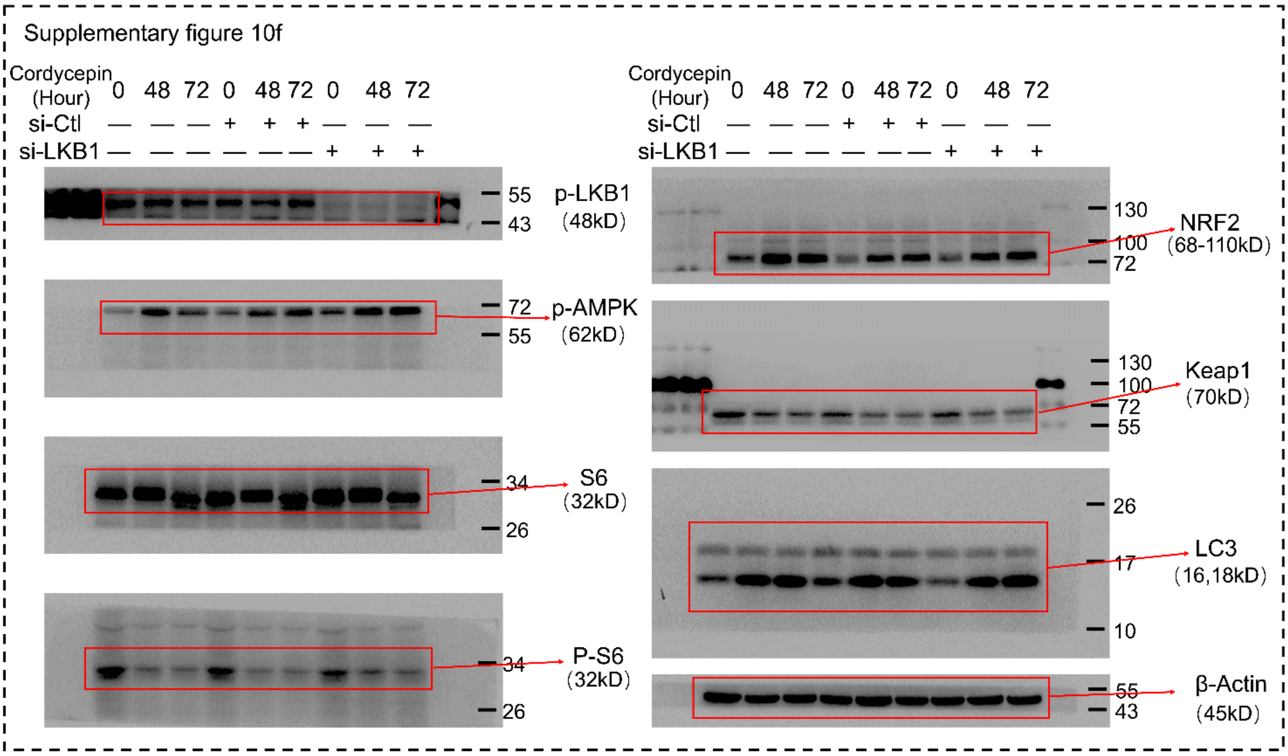
Supplementary figure 10f
Cordycepin (Hour) 0 48 72 0 48 72 0 48 72
si-Ctl — — — + + + — — —
si-LKB1 — — — — — — + + +
p-LKB1 (48kD)
55
43
p-AMPK (62kD)
72
55
S6 (32kD)
34
26
P-S6 (32kD)
34
26
NRF2 (68-110kD)
130
100
72
Keap1 (70kD)
130
100
72
55
LC3 (16,18kD)
26
17
10
β-Actin (45kD)
55
43

Supplementary Table 1. Sequences of the primers used for qRT-PCR

Gene	Forward sequences	Reverse sequences
Rat IL1β	5-GATGATGACGACCTGCTAGTGTGT-3	5-TTGGCTTATGTTCTGTCCATTGAG-3
Rat IL6	5-CTTCCAGCCAGTTGCCTTCTT-3	5-GGTCTGTTGTGGGTGGTATCCT-3
Rat TNFα	5-CTCATTCCTGCTCGTGGCG-3	5-CGTGGGCTACGGGCTTGT-3
Rat β-actin	5-CTGAGAGGGAAATCGTGCGT-3	5-AGGGAGGAAGAGGATGCGG-3
Mouse IL1β	5-TGCCACCTTTTGACAGTGATG-3	5-ATGTGCTGCTGCGAGATTTG-3
Mouse IL6	5-AGTCAATTCCAGAAACCGCTATGA-3	5-TAGGGAAGGCCGTGGTTGT-3
Mouse TNFα	5-TTCTCATTCCTGCTTGTG-3	5-TTGGTGGTTTGCTACG-3
Mouse CDKN2A	5-CGCAGGTTCTTGGTCACTGT-3	5-TGTTCACGAAAGCCAGAGCG-3
Mouse CDKN1A	5-CGAGAACGGTGGAACTTTGAC-3	5-CCAGGGCTCAGGTAGACCTT-3
Mouse β-actin	5-AACAGTCCGCCTAGAAGCAC-3	5-CGTTGACATCCGTAAAGACC-3
Human IL1α	5-AATGACGCCCTCAATCAAAG-3	5-TGGGTATCTCAGGCATCTCC-3
Human IL1β	5-ATGATGGCTTATTACAGTGGCAA-3	5-GTCGGAGATTCGTAGCTGGA-3
Human IL6	5-ACTCACCTCTTCAGAACGAATTG-3	5-CCATCTTTGGAAGGTTCAGGTTG-3
Human IL8	5-ACTGAGAGTGATTGAGAGTGGAC-3	5-AACCCTCTGCACCCAGTTTTC-3
Human MMP3	5-AGTCTTCCAATCCTACTGTTGCT-3	5-TCCCCGTCACCTCCAATCC-3
Human MMP12	5-GATCCAAAGGCCGTAATGTTCC-3	5-TGAATGCCACGTATGTCATCAG-3
Human PAI1	5-TGGGTGAAGACACACACAAAAGG-3	5-GTGCTGGAGTCGGGGAAGG-3
Human FOXO1	5-AACCTGGCATTACAGTTGGCC-3	5-AAATGCAGGAGGCATGACTACGT-3
Human FOXO3a	5-TCAATCAGAACTTGCTCCACCA-3	5-GGACTCACTCAAGCCCATGTTG-3
Human FOXO4	5-TTTTCTCACTGTGCCAATTAGGG-3	5-TCCAACAGCATTGCTCATCTTG-3
Human GSTA1	5-AGCCCAAGCTCCACTACTTCAAT-3	5-CTTCAAACTCTACTCCAGCTGCAG-3
Human Srx	5-GCCCAGGGAGGTGACTACTT-3	5-GTGGATGCTCCCAGGTACAC-3
Human NQO1	5-CGCAGACCTTGTGATATTCCAG-3	5-CGTTTCTTCCATCCTTCCAGG-3
Human NRF2	5-TCCAGTCAGAAACCAGTGGAT-3	5-GAATGTCTGCGCCAAAAGCTG-3
Human Keap1	5-CCTTCAGCTACACCCTGGAG-3	5-CATGACCTTGGGGTGGATAC-3
Human AMPKγ1	5-ATGAAGTCTCATCGCTGCTATG-3	5-ACCGTTAGTCACCAAAGCAAA-3
Human AMPKγ2	5-TGCCCGTTATTGACCCTATCA-3	5-CAGGCTTTGGCATATCAGACAT-3
Human AMPKγ3	5-CCTGGGAGTGTGAGCTAGAAG-3	5-GTTTCCGCAGTTCGTCATCC-3
Human AMPKα1	5-GGCACGCCATACCCTTGAT-3	5-TCTTCCTTCGTACACGCAAATAA-3
Human AMPKα2	5-CTGTAAGCATGGACGGGTTGA-3	5-AAATCGGCTATCTTGGCATTCA-3
Human GAPDH	5-GGAGCGAGATCCCTCCAAAAT-3	5-GCTGTTGTCATACTTCTCATGG-3

Supplementary Table 2. Sequences of the siRNAs for RNA interference

Gene	Forward sequences	Reverse sequences
NRF2	5-CCUGCUACUUUAAGCCAUUTT-3	5-AAUGGCUUAAAGUAGCAGGTT-3
P62	5-GGAGUCGGAUAACUGUUCATT-3	5-UGAACAGUUAUCCGACUCCTT-3
Atg7	5-GGAUCCUGGACUCUCUAAATT-3	5-UUUAGAGAGUCCAGGAUCCTT-3
LKB1	5-GGAUGUGUUAUACAACGAATT-3	5-UUCGUUGUAUAACACAUCCTT-3
AMPKγ1	5-GAGGAGAGCUAUUUGAUUATT-3	5-UAAUCAAAUAGCUCUCCUCTT-3
AMPKα1	5-CUGCACCGCUACUAUAAAUTT-3	5-AUUUAUAGUAGCGGUGCAGTT-3
NC siRNA	5-UUCUCCGAACGUGUCACGUTT-3	5-ACGUGACACGUUCGGAGAATT-3
